# A genetically-encoded crosslinker screen identifies SERBP1 as a PKCε substrate influencing translation and cell division

**DOI:** 10.1038/s41467-021-27189-5

**Published:** 2021-11-26

**Authors:** Silvia Martini, Khalil Davis, Rupert Faraway, Lisa Elze, Nicola Lockwood, Andrew Jones, Xiao Xie, Neil Q. McDonald, David J. Mann, Alan Armstrong, Jernej Ule, Peter J. Parker

**Affiliations:** 1 Protein Phosphorylation Laboratory, The Francis Crick Institute, London, UK; 2 RNA Network Laboratory, The Francis Crick Institute, London, UK; 3 Department of Neuromuscular Diseases, UCL Queen Square Institute of Neurology, Queen Square, London, WC1N 3BG UK; 4 Department of Human Genetics, Radboud University Medical Center, Nijmegen, The Netherlands; 5 Cell Cycle Laboratory, The Francis Crick Institute, London, UK; 6 Synthetic and Functional Biomolecules Center, Beijing National Laboratory for Molecular Sciences, College of Chemistry and Molecular Engineering, Peking University, Beijing, China; 7 Signalling and Structural Biology Laboratory, The Francis Crick Institute, London, UK; 8 Department of Life Sciences, Imperial College London, London, SW7 2AZ UK; 9 Department of Chemistry, Imperial College, London, UK; 10 School of Cancer and Pharmaceutical Sciences, King’s College London, Guy’s Campus, London, UK

**Keywords:** Stress signalling, Chromosome segregation, Mitosis

## Abstract

The PKCε-regulated genome protective pathway provides transformed cells a failsafe to successfully complete mitosis. Despite the necessary role for Aurora B in this programme, it is unclear whether its requirement is sufficient or if other PKCε cell cycle targets are involved. To address this, we developed a trapping strategy using UV-photocrosslinkable amino acids encoded in the PKCε kinase domain. The validation of the mRNA binding protein SERBP1 as a PKCε substrate revealed a series of mitotic events controlled by the catalytic form of PKCε. PKCε represses protein translation, altering SERBP1 binding to the 40 S ribosomal subunit and promoting the assembly of ribonucleoprotein granules containing SERBP1, termed M-bodies. Independent of Aurora B, SERBP1 is shown to be necessary for chromosome segregation and successful cell division, correlating with M-body formation. This requirement for SERBP1 demonstrates that Aurora B acts in concert with translational regulation in the PKCε-controlled pathway exerting genome protection.

## Abstract (web summary)

PKCε is known to exert a role in genome protection by directly phosphorylating and switching the specificity of Aurora B. Here the authors identify SERBP1 as a parallel mitotic PKCε substrate controlling translation and ensuring the integrity of chromosome segregation and successful cell division.

## Introduction

Protective measures are engaged extensively during cell cycle progression to ensure the integrity of genome duplication and the fidelity of the ensuing daughter cells genetic inheritance. A significant role has emerged recently for PKCε in controlling protection from chromosome nondisjunction and successful cell division^1,2^, specifically in a subset of transformed cells that fail to arrest in G2 phase in response to the Topoisomerase 2 (Topo2) inhibitor ICRF-193^3^. Studies to date indicate that PKCε exerts this role in genome protection by directly phosphorylating and switching the specificity of Aurora B^4,5^. However, the complex interplay of kinases and associated regulatory inputs to cell cycle control, begs the question of whether this PKCε-Aurora B relationship is both necessary and sufficient or whether there are in fact other cell cycle targets of PKCε that contribute to these protective mechanisms. It has recently been established that PP1 recruitment through its binding partner RIF1, contributes to the timing of the Aurora B abscission checkpoint exit^6^, working in concert with PKCε^5^ and providing evidence for multiple controls acting at this juncture and underscoring the need to address the multiplicity of actions of these regulators.

Although technological advances allow the identification of multiple phosphorylation sites simultaneously, it is still remarkably difficult to definitively link a particular substrate phosphorylation event with a single upstream kinase in a cellular context. Here we exploited genetic code expansion technology for UV-induced substrate crosslinking. We employed the PKC-substrate structure^7^ to select the residues for the insertion of a photo-cross-linkable amino acid in the PKCε kinase domain, in proximity to the substrate-binding pocket; UV irradiation allowed the PKCε catalytic domain crosslinking with potential substrates. Among the candidates identified, we investigated SERPINE1 mRNA Binding Protein 1 (SERBP1), an arginine-methylated mRNA binding protein, which was initially described as binding the 3’-UTR of type-1 plasminogen activator inhibitor (PAI-1) mRNA, and hence regulating its stability^8^.

We validated SERBP1 as a genuine PKCε substrate by demonstrating that: (i) SERBP1 is phosphorylated by PKCε in vitro (ii) that the main site of phosphorylation is S74 and (iii) that this is the major site modified by PKCε in cells. Moreover, with respect to functional consequences, we demonstrate that: (i) in mitosis, SERBP1 accumulates in RNA-containing mitotic structures (M-bodies, identified and characterised here) under PKCε control, requiring S74 phosphorylation, (ii) PKCε controls SERBP1 binding to ribosomes during M-phase, resulting in suppression of translation (iii) that SERBP1 is required for genome integrity and cell division, a component of the protective pathway regulated by the M-phase cleaved form of PKCε. With the demonstration of complete independence of these events from Aurora B, we determine that PKCε controls multiple M-phase events that collectively contribute to protect cells from division failure.

## Results

### A screen for PKCε substrates involved in cell division

**Fig. 1:**
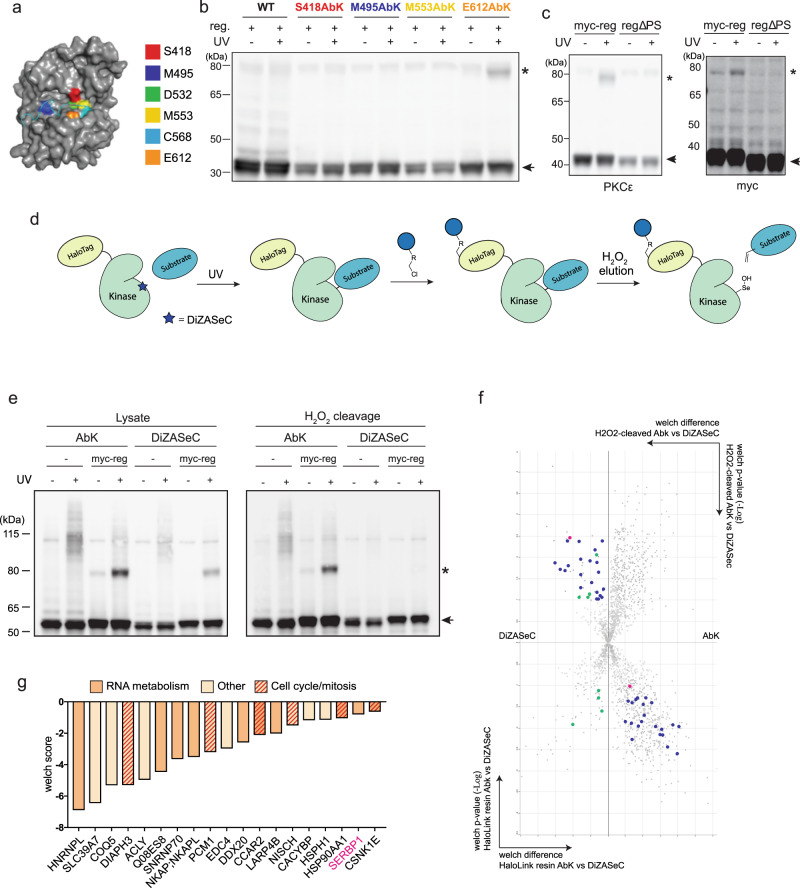
A genetically encoded cross-linker screen identifies SERBP1 as a PKCε substrate. In all blots, asterisks indicate crosslinked PKC catalytic domain and arrows the free catalytic domain. **a** Homology model of PKC catalytic domain bound to a peptide substrate based upon PKCι bound to a Par3 peptide crystal structure (PDB:4DC2). Colours indicate the six residues selected for the Abk insertion. **b** WT or TAG mutant catalytic domain expressed in HEK293T cells with myc-tagged PKCε regulatory domain (reg.), AbK incorporation, UV irradiated or not, blotted for PKCε. **c** PKCε E612TAG catalytic domain and myc-tagged PKCε regulatory domain either wildtype (reg) or without the pseudosubstrate sequence (reg∆PS) co-expressed under AbK incorporation, blotted for PKCε (left) and myc (right). **d** Scheme representing the cross-linking protocol employing the PKCε E612AbK with an N-terminal HaloTag followed by UV irradiation. **e** HaloTag-PKC E612TAG expressed in cells under AbK or DiZASeC incorporation conditions. Where indicated, myc-tagged PKCε regulatory domain was co-expressed. **f** Volcano plot showing both the H_2_O_2_-cleaved (upper volcano plot) and protein remaining on the HaloLink resin (inverted, lower plot) after cleavage of DiZASeC and AbK. The *X*-axis represents welch difference, with a negative value more enriched in the DiZASeC sample, and a positive value more enriched in the AbK sample. The *Y*-axis represents log welch p-value, with a higher value (or more negative value for the inverted plot) signifying higher confidence in the hit. In blue, hits significantly enriched for DiZASeC in the H_2_O_2_-cleaved condition (upper-left quadrant) and for the AbK condition on the HaloLink resin (lower-right quadrant). In green, hits enriched in the DiZASeC condition in the HaloLink resin. In grey, hits with no significant enrichment in the HaloLink resin condition. SERBP1 is indicated in magenta. **g** Graph shows potential PKCε substrates identified in (**f**), distributed based on the welch score. Proteins involved in RNA binding and/or cell cycle/mitosis are indicated as described (see Supplementary Table 1). Source data are provided as a Source Data file.

To identify candidate cell cycle substrates for PKCε, we exploited the kinase domain previously shown to be generated proteolytically in M-phase^4^. Using a genetic code expansion approach we incorporated the photo-cross-linkable aminoacid Abk^9^ into the kinase domain proximal to the substrate-binding pocket, with the intention of crosslinking interacting proteins upon UV-irradiation (Supplementary Fig. 1a). In full-length, autoinhibited PKC, the kinase domain interacts with the regulatory domain through a pseudosubstrate interaction^10^, hence we tested the ability of AbK modified kinase domain to crosslink the regulatory domain, following co-expression of the individually encoded domains in HEK293T cells. Based on the PKCι-Par3 structure^7^, we selected six residues for the introduction of amber suppressor mutations, enabling AbK insertion at these positions (Fig. 1a). Four of the six mutants showed phosphorylation at PKCε ‘priming’ sites, T566 and S729, indicating that the insertion of AbK was tolerated without grossly affecting protein folding and catalytic competency (Supplementary Fig. 1b). Upon UV irradiation of these four variants co-expressed with the regulatory domain, the E612AbK mutant uniquely led to the specific appearance of a PKCε antigen at ~78KDa, consistent with covalent crosslinking to the co-expressed myc-tagged regulatory domain (Fig. 1b). The use of the nonphotoactive mutant E612BocK did not give rise to this band, confirming that the crosslinking of these two species occurred through AbK (Supplementary Fig. 1c). Importantly, the PKCε regulatory domain where the pseudosubstrate sequence was deleted (reg ∆PS), was not crosslinked by E612AbK catalytic domain upon UV irradiation, indicating that substrate-site docking of the regulatory domain is necessary for the crosslinking to occur (Fig. 1c). To assess whether PKCεE612AbK catalytic domain retains substrate-binding capacity and has the same substrate-specificity as the WT, we confirmed that levels on the priming phosphorylation sites T566, T710 and S729 and the pattern of induced phosphorylation of endogenous proteins were comparable to the WT (Supplementary Fig. 1d). We then investigated the ability of the PKCεE612AbK catalytic domain to crosslink to endogenous substrates. UV irradiation of cells expressing PKCεE612AbK catalytic domain in the absence of the regulatory domain gave rise to a number of bands that potentially represented PKCε crosslinked to substrates (Supplementary Fig. 1e). PKCεE612AbK was then isolated from both UV-treated and untreated cells and protein content compared by mass spectrometry. Despite the substantial background observed, the RNA-binding protein SERPINE1 mRNA binding protein 1 (SERBP1) was identified as being significantly enriched in the UV-treated sample (Supplementary Fig. 1f).

To optimise the substrate screening process and reduce the background, we used an irreversible HaloTag purification approach combined with the peroxide-cleavable amino acid DiZASeC^11^. We generated HaloTag-PKCε with E612DiZASeC to irreversibly trap crosslinked complexes on HaloLink resin; this enabled subsequent stringent washing followed by specific release of crosslinked proteins by treating the resin with H_2_O_2_ (Fig. 1d). HaloTag-PKCεE612AbK and E612DiZASeC catalytic domains both crosslinked to PKCε regulatory domain and endogenous proteins. As expected, treatment of these crosslinked complexes with H_2_O_2_ led to crosslink cleavage only in the case of E612DiZASeC (Fig. 1e). Comparing protein abundance by mass spectrometry for proteins cleaved from the resin and those remaining bound to the beads in AbK and DiZASeC conditions, enabled the identification of a subset of proteins that were enriched in the released condition for DiZASeC and retained on the beads for AbK, suggesting these represented genuine targets (Fig. 1f). As well as a series of other proteins, this approach confirmed SERBP1 as a hit (Fig. 1g, Supplementary Table 1).

### SERBP1 is a PKCε substrate in vitro and in cells

**Fig. 2:**
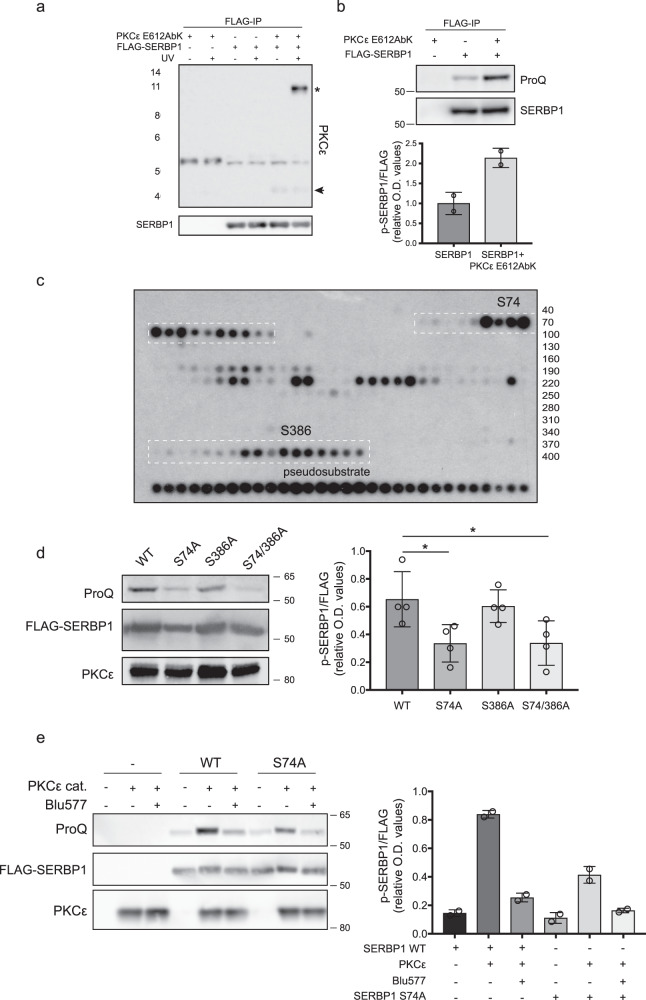
SERBP1 is phosphorylated at Serine 74 (S74) both in vitro and in cells. **a** Cells were irradiated with 365 nm UV light and lysed, followed by immunoprecipitation of FLAG-SERBP1 using magnetic beads coupled to an anti-FLAG antibody. Immunoprecipitates (FLAG-IP) were analysed by western blot with PKCε and SERBP1 antibodies. The arrow indicates the catalytic domain of PKCε and the asterisk a cross-linked species of PKCε (**b**) FLAG-SERBP1 was expressed in HEK293T cells, with or without PKCε E612AbK as indicated. Extracts were subjected to FLAG-immunoprecipitation. Phosphorylation levels of immunoprecipitated FLAG-SERBP1 were then determined using the ProQ diamond phosphostain (ProQ, upper panel); mean and range of *n* = 2 independent experiment. **c** Peptide array showing S74 and S386/T388 as potential phosphorylation sites. The array comprised 20mers with a single amino acid step between each, starting top left with residues 1–20, then 2–21, etc. The bottom line of the array was populated with an optimised PKCε pseudosubstrate sequence as a positive control. The image is representative of one experiment carried out in technical duplicate. Note single ‘hot’ spots or short runs are considered non-specific. (**d**, left) ProQ analysis of FLAG-pulled down SERBP1 WT, S74A, S386A and S74/386A transfected 293 T cells. (**d**, right) Quantification of the ProQ diamond staining normalised on the FLAG expression. Error bars, mean ± SD of *n* = 4 independent experiments, One way-ANOVA and Tukey’s multiple comparisons test, *p = 0.03, ns = 0.94, *p = 0.04. (**e**, left) ProQ analysis of immunoprecipitated FLAG-SERBP1 WT or the S74A mutant from transfected 293 T cells. Immunocomplexed FLAG-SERBP1 WT or S74A were exposed to purified PKCε WT catalytic domain (PKCε cat.) in kinase assay buffer containing Mg-ATP with, or without, the presence of 500 nM PKCε inhibitor Blu577 as indicated. Images are representative of 3 independent experiments. (**e**, right) Changes in phosphorylation are quantified for all experiments under the conditions indicated; mean and range of *n* = 2 independent experiment. Source data are provided as a Source Data file.

As SERBP1 was found in both crosslinking approaches, we investigated whether this protein represented a meaningful hit. We co-expressed tagged SERBP1 with the PKCεE612AbK catalytic domain and found that SERBP1 became crosslinked to PKCεE612AbK on UV exposure, as evident from the PKCε immunoreactive species of 115KDa recovered in the SERBP1 immunocomplex (Fig. 2a). In addition, co-expression of PKCε increased the overall SERBP1 phosphorylation, indicating that SERBP1 is a PKCε substrate (Fig. 2b). To assess candidate site(s) of phosphorylation by PKCε, we employed a custom peptide array covering the coding sequence of SERBP1 in 20mers with a single amino acid step between peptides. This led to the identification of two sites: a major site at Serine 74 (S74), which has the characteristic basophilic nature of a PKC site (KQLRKESQKDRKN) and a second weaker site at Serine 386 (S386) (Fig. 2c). We generated FLAG-SERBP1 constructs with the relevant Ser to Ala mutations and co-expressed them with the PKCε kinase domain in HEK293T cells. Although S386A mutation did not affect SERBP1 phosphorylation, S74A and S74/386 A mutants displayed significantly reduced SERBP1 phosphorylation levels, confirming S74 as the dominant phosphorylation site under PKCε control in cells (Fig. 2d). We validated this finding through an in vitro kinase assay using purified PKCε catalytic domain and immune-precipitated FLAG-SERBP1, either in the presence or absence of the novel PKC inhibitor Blu577^12^ (Fig. 2e). Although the S74 mutant is phosphorylated to some extent by recombinant PKCε, the total level of phosphorylation is significantly lower compared to SERBP1 WT. The addition of Blu577 resulted in reduced phosphorylation also in the S74A mutant suggesting that additional PKC sites may be present. Combined, these data confirm that SERBP1 S74 is a genuine substrate of PKCε.

### PKCε regulates SERBP1 accumulation in mitotic structures (M-bodies)

**Fig. 3:**
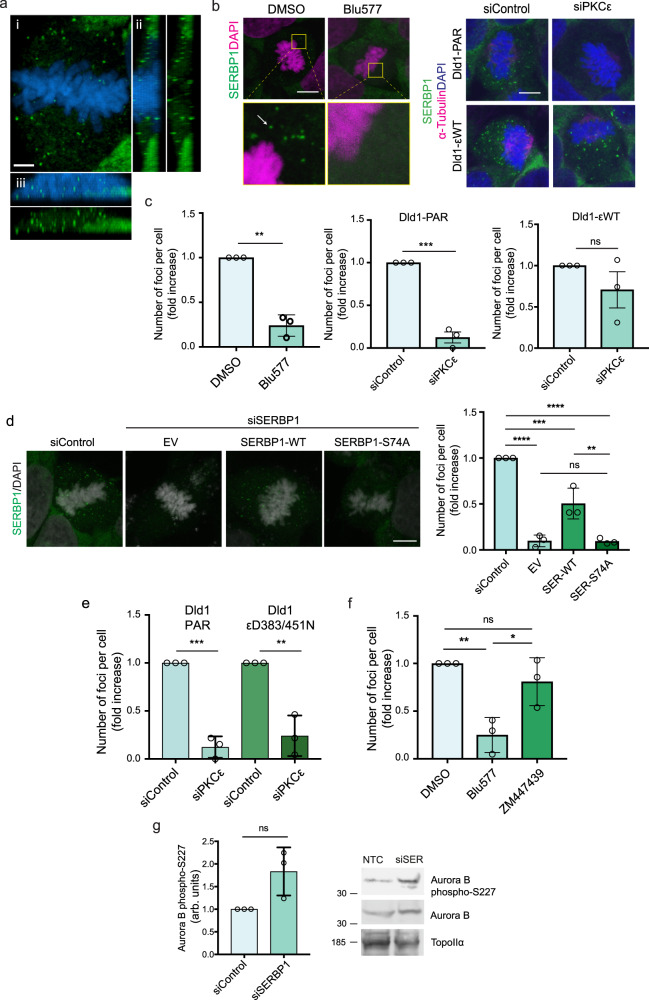
SERBP1 accumulates in mitotic structures (M-bodies) in a PKCε-dependent and Aurora B-independent manner. **a** SERBP1^+^ M-bodies are detected in green and DNA in blue. Scale bar, 10 μm. Panel (i) is a projected image of M-bodies (green) at the metaphase plate (DNA in blue) and in (ii) and (iii) orthogonal z-plane sections along and across the metaphase plate with both the M-body image and a combined image with DAPI; see also Supplementary Movie 1. (**b**, left) SERBP1 (green) localisation in cells treated with DMSO or with 500 nM Blu577 for 1 h. Scale bar, 10 μm. DNA is shown in magenta. Arrow indicates SERBP1^+^ M-bodies. (**b**, right) DLD1 parental cells (DLD1-PAR) and DLD1 cells expressing GFP-PKCε WT (DLD1-εWT), transfected with siRNA Non-targeting control (siControl) and specific siRNA targeting PKCε (siPKCε). SERBP1 in green, α-Tubulin in magenta and DAPI in blue. Scale bar, 10 μm. **c** Quantification of the number of M-bodies/cell ± Blu577 treatment (left), in DLD1-PAR or DLD1-εWT cells transfected with siControl or siPKCε (right). *n* = 15 cells examined over three independent experiments. Error bars, mean ± SD. Unpaired *t* test, ***p = 0.0004 DMSO vs Blu577, ***p = 0.0002 DLD1-PAR siControl vs siPKCε, ns = 0.25 DLD1-εWT siControl vs siPKCε. **d** Cells transfected with siControl or siSERBP1, transiently expressing the empty vector (EV), FLAG-SERBP1-WT and FLAG-SERBP1-S74A. SERBP1 in green and DAPI in grey. Scale bar, 10 μm. (**d**, right) Quantification of SERBP1 M-bodies. *n* = 15 cells examined over three independent experiments. Error bars, mean ± SD. One way-anova, Tukey’s multiple comparisons test, ****p < 0.0001, ***p = 0.0007, **p = 0.002, ns = non-significant. **e** M-body quantification in siControl and siPKCε-transfected DLD1 parental and DLD1-εD383/451 N cells. *n* = 20 cells examined in three independent experiments. Error bars, mean ± SD. Unpaired *t* test, **p = 0.0002, **p = 0.003. **f** M-bodies quantification in cells treated with DMSO, 500 nM Blu577 or 2μM ZM447439, for 1 h. *n* = 15 cells examined in 3 independent experiments. Error bars, mean ± SD. One way-ANOVA, Tukey’s multiple comparisons test, *p = 0.02, **p = 0.005, ns = 0.45. **g** Quantification of Aurora B phospho-S227 normalised on Aurora B. Error bars, mean ± SD of *n* = 3 independent experiment. Unpaired *t* test, ns = 0.15. On the right, representative blots of phospho-S227 Aurora B and total Aurora B. Source data are provided as a Source Data file.

SERBP1 subcellular compartmentalisation, which can be regulated by PRMT1‐mediated arginine methylation^13^, is important in the expression of its function. In the nucleus, SERBP1 is involved in chromatin remodelling and transcription^14,15^, while in the cytoplasm it regulates mRNA stability through its association with ribosomes^16^ and stress granules^17^. We observed SERBP1 diffuse in the cytoplasm of interphase DLD1 cells. However, analysis of mitotic cells revealed a previously unreported accumulation of SERBP1 in foci ranging in size from 0.1 to 0.5 μM (Fig. 3a; Supplementary Video 1). Unlike structures such as stress granules and processing bodies (P-bodies), which are known to disassemble when cells enter mitosis^18,19^, the cytoplasmic SERBP1^+^ foci described here are formed and dynamically maintained exclusively from prometaphase to cytokinesis (Supplementary Fig. 2a). A similar M-phase dependent localisation of SERBP1 was also observed in HeLa and HEK293T cells (Supplementary Fig. 2b). To assess if SERBP1^+^ cytoplasmic foci formation is strictly a mitotic event, we synchronised DLD1 cells in mitosis, performed a mitotic shake-off and then re-seeded cells separately to observe whether the foci were retained after completion of cytokinesis (Supplementary Fig. 2c). No foci were identified in the new interphase, indicating that these are indeed transient M-phase structures. Therefore, we refer to the SERBP1^+^ cytoplasmic foci as SERBP1 mitotic-bodies (M-bodies).

To investigate whether M-bodies are dependent on PKCε, we analysed mitotic cells treated with Blu577 or after knock-down of endogenous PKCε. We observed a significant reduction in the number of SERBP1^+^ M-bodies (Fig. 3b, c), without affecting SERBP1 protein levels (Supplementary Fig. 2d), suggesting that PKCε is specifically involved in the regulation of M-body formation. This was confirmed by the significant rescue of mitotic foci observed in DLD1 cells stably expressing siRNA-resistant GFP-PKCε WT (Fig. 3b, c, Supplementary Fig. 2e). Knock down of the endogenous SERBP1 followed by transient expression of siRNA resistant FLAG-SERBP1 WT or the FLAG-SERBP1 S74A mutant did not result in M-body rescue when S74 cannot be phosphorylated (Fig. 3d, Supplementary Fig. 2f). This indicates that PKCε phosphorylation of SERBP1 at S74 is required for SERBP1^+^ M-body formation.

In mitosis, PKCε cleavage by Caspase 7 at D383 induces the release of a pool of constitutively active kinase domain to facilitate chromosome non-disjunction resolution and successful cell division^4,20^. To assess whether M-body formation was controlled by this mitotic pathway, we investigated M-body formation in DLD1 cells stably expressing PKCε where cleavage was prevented by D383/451N mutation (Fig. 3e, Supplementary Fig. 2g)^4^. This mutant was unable to rescue M-body formation on endogenous PKCε knockdown. With M-body formation positioned on this PKCε mitotic pathway, we determined whether these structures were directly linked to Aurora B also downstream of PKCε. Acute inhibition of Aurora B activity using the inhibitor ZM447439 demonstrated that M-body formation was not downstream of Aurora B signalling (Fig. 3f). Reciprocally, we found that PKCε phoshorylation sites on Aurora B and Topoisomerase 2α necessary for catenation resolution during mitosis, were not affected by SERBP1 knockdown (Fig. 3g, Supplementary Fig. 2h). Collectively, the data indicate that M-body formation is a mitotic event controlled by the Caspase7-PKCε pathway independently of Aurora B function and not required for Aurora B S227 phosphorylation.

### Properties of SERBP1 M-bodies

**Fig. 4:**
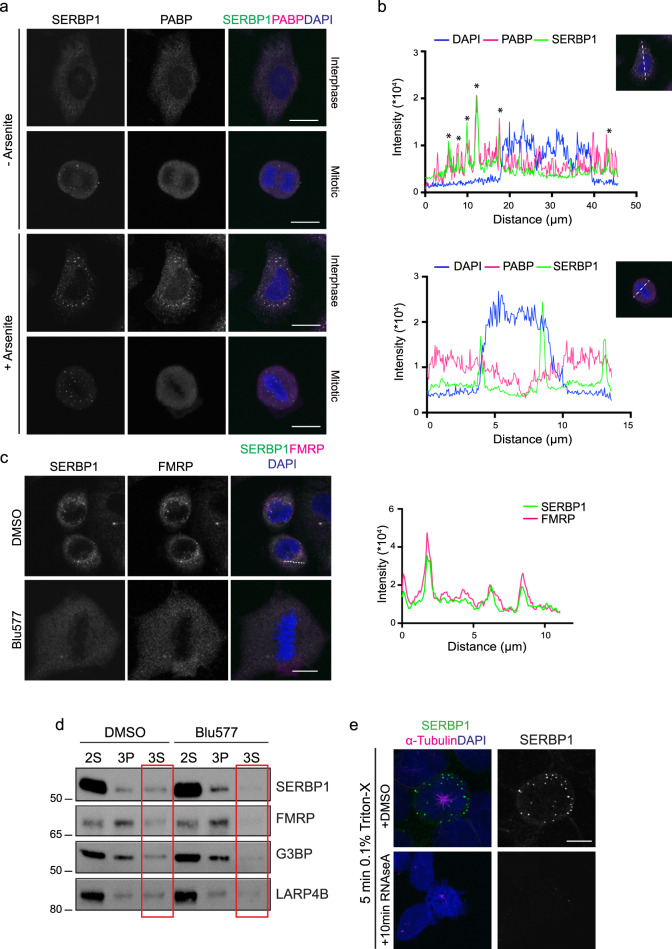
SERBP1 M-bodies are distinct from the canonical stress granules. **a** DLD1 cells were treated with or without 0.5 mM arsenite for 30’ min. Cells in interphase or mitotic cells (as indicated) were stained with anti-SERBP1 (green), anti-PABP (magenta) and DAPI (blue). Scale bar, 10 μm. **b** PABP (magenta) and SERBP1 (green) colocalisation profile in arsenite-treated interphase cell (top) and arsenite-treated mitotic cell (bottom). DAPI profile is in blue. Graphs show the fluorescence intensity picked on the dotted white line, asterisks indicate SERBP1/PABP colocalisation. **c** Representative mitotic DLD1 cells treated or not with 500 nM Blu577 for 1 h. Cells were stained with SERBP1 (green), FMRP (magenta) and DAPI (blue). Scale bar, 10 μm. On the right, the plot shows the fluorescence profile of SERBP1 (green) and FMRP (magenta) picked on the white line shown in the representative image. **d** Fractionation assay in mitotic enriched DLD1 cells, treated with or without 500 nM Blu577 for 1 h. The second supernatant (2 S), third pellet (3 P) and third supernatant (3 S) were immunoblotted for LARP4B, FMRP, G3BP and SERBP1, which are modulated by Blu577 in the 3 S fraction, as indicated by the red rectangles. **e** DLD1 cells permeabilised for 5 min with 0.1% Triton-X prior to 10 min RNaseA treatment. SERBP1 M-bodies in green, α-Tubulin in magenta and DNA in blue (DAPI). Scale bar, 10 μm. Source data are provided as a Source Data file.

Under specific adverse environmental conditions, SERBP1 is known to accumulate in stress granules (SGs); these dynamic non-translating RNA protein assemblies form to sequester/protect mRNA^21^. Although it has been demonstrated that mitotic cells are resistant to SG assembly^18^, we speculated that the SERBP1^+^ M-bodies might be cytoplasmic SGs specifically expressed in mitosis. As expected, arsenite treatment induced SG formation in interphase cells (Fig. 4a) resulting in large cytoplasmic granules containing SERBP1 and the SG marker PABP. However, these structures and the colocalization of SG proteins were lost when cells were in mitosis (Fig. 4b). Despite showing a punctate staining pattern in mitotic cells, the 40 S ribosomal component RPS6 and the P-body marker Ago2 were not found enriched in the M-bodies (Supplementary Fig. 3a), indicating their distinctiveness. However, we found that the Fragile X Mental Retardation Protein (FMRP), reported previously to interact with SERBP1 in stress granules^17,22^, localised to the M-bodies in a manner dependent on both PKCε and SERBP1 expression (Fig. 4c, Supplementary Fig. 3b). The loss of these FMRP^+^ structures on knockdown of SERBP1 indicates that these likely do not form unless SERBP1 is present. Reciprocally, FMRP proved necessary for M-body formation and or/maintenance since its downregulation resulted in loss of SERBP1 foci (Supplementary Fig. 3b).

Microtubule-associated proteins such as dyneins and kinesins are critical for SG assembly and movement. Given the role of PKCε in centrosome movement and mitotic spindle assembly through dynein interaction^23^, we investigated M-body dynamics upon induced disruption of mitotic spindles. Unlike stress granules, neither Dynein nor Eg5 inhibition using EHNA, STLC and Ciliobrevin affected the M-bodies (Supplementary Fig. 3c), indicating that these microtubule motor proteins are not involved in M-body formation.

We further characterised M-body composition using a purification method previously described for isolation of SG cores from mammalian cells^24,25^. We validated the method (described in Supplementary Fig. 3d) by treating cells with arsenite and using immunoblotting to analyse the three fractions obtained: plasma membranes and cytoplasmic proteins (2 S), a detergent-insoluble organelle extract (3 P) and a soluble organelle extract (3 S). We observed SERBP1 mainly enriched in the 3 S fraction obtained from arsenite-treated samples. The presence of G3BP, known to trigger SGs primary aggregation, confirmed the 3 S fraction as the one containing the stress granule cores (Supplementary Fig. 3e). We then adapted this method to cells synchronised in mitosis and isolated through mitotic shake-off, with or without Blu577. To better characterise the fractionation assay applied, we observed the expression of the known membrane, cytoplasm, organelle and ribosomal proteins such as EGFR, Vimentin, GAPDH, GM130 and RPS6 (Supplementary Fig. 3f). These proteins were found enriched in the 2 S and 3 P fractions and were not affected by PKCε inhibition. In the 3 S fraction, normalised to the sum of all fractions, both SERBP1 and FMRP levels are reduced upon PKCε inhibition (band intensity from 3.2 to 0.5 and from 29 to 10, respectively). Unexpectedly, we observed a similar reduction of G3BP levels in the same fraction (band intensity from 9 to 3.5), indicating that G3BP may be contained in M-bodies and modulated by PKCε (Fig. 4d).

Alongside SERBP1 and FMRP, the mRNA binding protein LARP4B is known to accumulate in stress granules^26^ and it has been identified as a potential PKCε substrate in our screen (Fig. 1g). Although we were unable to detect LARP4B in SERBP1^+^ M-bodies by IF with available antibodies, by western its expression appeared in the same fraction as the M-bodies and was reduced by Blu577 treatment similarly to SERBP1 and FMRP (band intensity from 12 to 6.3 in the 3 S fraction; Fig. 4d), suggesting it may also be a component of these structures.

Finally, given the known role of both SERBP1 and FMRP in RNA stability and translation^16,27^, we assessed whether the M-bodies contain RNA. DLD1 cells permeabilized with 0.1% Triton for 1 or 5 min were treated with RNaseA for 10 minutes (Fig. 4e, Supplementary Fig. 3h). One-minute permeabilization is enough to observe a reduction of SERBP1^+^ M-bodies as a function of RNaseA treatment; these disappear completely from the cytoplasm when cells are permeabilised for longer, indicating that the M-bodies are RNA-dependent structures and by inference like SGs have the potential to influence translation.

### SERBP1 ribosomal binding in mitosis is selectively modulated by PKCε

**Fig. 5:**
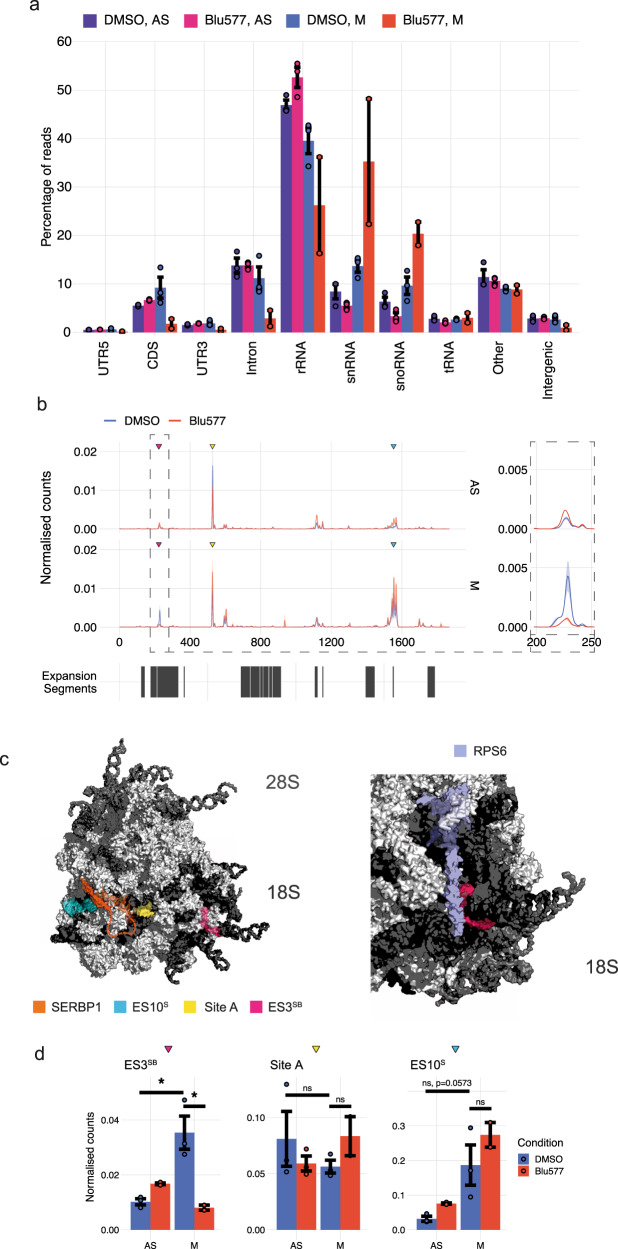
PKCε affects SERBP1 ribosomal binding in cells enriched in mitosis. **a** The mean proportion of SERBP1 iCLIP reads aligning to different subtypes of RNA in asynchronous (AS) and mitotic (M) conditions following 2-h treatment with DMSO or 500 nM Blu577. Error bars, mean ± SD of *n* = 3 independent experiments. Welch two-sample *t* test. Two independent experiments for cells treated in mitosis with Blu577. **b** Metaprofiles of iCLIP crosslinking to 18S rRNA. Lines represent mean values for samples within that condition, while shaded regions represent the standard error. Normalised counts are the count of crosslinks at a position normalised by the number of rRNA reads for a given sample. The boxed area is expanded to the right to illustrate the M-specific effects of BLU577 (**c**) Illustration of SERBP1 iCLIP crosslinking positions on the 80S ribosome structure^28^. **d** Quantification of normalised SERBP1 iCLIP counts within ES3SB (200–250nt), Site A (510–550nt) and ES10S (1500–1600nt). Error bars, mean ± SD of *n* = 3 independent experiment. Welch two-sample *t* test. Two independent experiments for cells treated in mitosis with Blu577. Source data are provided as a Source Data file.

In the cytoplasm, SERBP1 is bound to the 40 S ribosomal subunit^28^ as well as to mRNA^8^. To determine if SERBP1-RNA interactions change in M-phase under PKCε control, we performed iCLIP^29^ on SERBP1 in asynchronous and mitotic cell populations, in the presence or absence of Blu577. Consistent with previous observations of SERBP1 spanning the 40S ribosomal subunit mRNA entry channel from the solvent-exposed side to the decoding centre^16,28^, we identified a large proportion of SERBP1 iCLIP reads mapping to ribosomal RNA (Fig. 5a). Analysis of SERBP1 crosslinks on 18S ribosomal RNA revealed multiple binding sites for SERBP1 (Fig. 5b). In asynchronous samples, SERBP1 crosslinks most strongly to position 527 of 18S, corresponding to the mRNA entry channel, in agreement with the cryo-EM structure of the ribosome (Fig. 5c, Site A; yellow)^28^. We identified a single binding peak in 18S rRNA that showed a significant change when comparing asynchronous and mitotic cells, mapping to a position in expansion segment (ES) 3SB that is located close to the N-terminus of RPS6. SERBP1 crosslinking at this position is 3.5-fold stronger in mitosis (*p* < 0.05), and this increase in binding was completely lost following treatment with Blu577 (Fig. 5d). No other captured interactions were sensitive to BLU577. SERBP1 binding around ES10S increased in mitosis, although this did not achieve significance (Fig. 5d). Taken together, we found that PKCε promotes mitotic ribosomal binding of SERBP1 close to the N-terminus of RPS6, away from the mRNA channel, thereby likely altering its function in the ribosome. This function is likely mediated by direct phosphorylation of SERBP1, since no difference in RPS6 phosphorylation was detected upon PKCε inhibition (1.16 ± 0.03 DMSO vs 1.10 ± 0.2 Blu577).

### PKCε represses mitotic protein translation

**Fig. 6:**
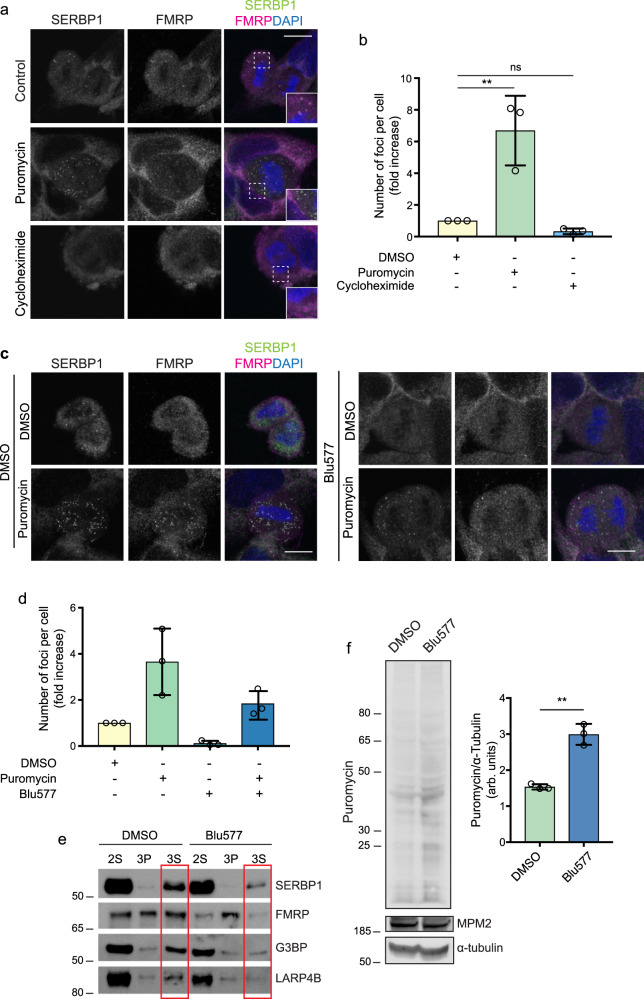
PKCε regulates protein translation in mitotic cells. **a** DLD1 cells treated for 1 hour with DMSO, 10 μg/ml cycloheximide or 20 μg/ml puromycin. SERBP1 is detected in green, FMRP in magenta and DNA in blue (DAPI). Scale bar, 10 μm. **b** Quantification of M-bodies per cell under the conditions described. *n* = 15 cells examined in three independent experiments. Error bars, mean ± SD. One way-ANOVA and Tukey’s multiple comparisons test, **p = 0.003, ns = 0.75. (**c**) After 1-hour treatment with DMSO or 500 nM Blu577, 20μg/ml puromycin was added for 1 hour to DLD1 cells. SERBP1 M-bodies are detected in green, α-Tubulin in magenta and DNA in blue (DAPI). Scale bar, 10 μm. **d** Number of M-bodies per cell are quantified under the conditions indicated. *n* = 15 cells examined over 3 independent experiments. Error bars, mean ± SD. **e** Western blot analysis of 2 S (second supernatant), 3 P (third pellet) and 3 S (third supernatant) fractions obtained from DLD1 treated for 1 h with DMSO or 500 nM Blu577 and then treated 1-h with 20 μg/ml puromycin. Samples were immunoblotted for LARP4B, FMRP, G3BP and SERBP1. The 3 S fraction containing the M-bodies is indicated by the red rectangles. **f** Ribopuromycylation assay in DLD1 cells synchronised in mitosis, isolated following mitotic shake off, treated with 50 μg/ml Puromycin and 100 μg/ml Cycloheximide for 5 min and blotted using the anti-Puromycin antibody. MPM2 staining is used to confirm mitotic enrichment and α-Tubulin is used as loading control. On the right, quantification of puromycin expression in DMSO- or 500 nM Blu577-treated mitotic cells for 1 h. α-tubulin has been used as a housekeeping control. Error bars, mean ± SD of *n* = 3 independent experiment. Unpaired *t* test, **p = 0.001. Source data are provided as a Source Data file.

The observation of a mitosis-specific, PKCε regulated change in SERBP1-40S interaction, mirrors the appearance and disappearance of M-bodies, and poses the question of the relationship between these; does a change in translation release mRNA for sequestration in M-bodies? To address the potential role of the M-bodies in such a response, we investigated puromycin and cycloheximide effects on the SERBP1/FMRP structures. Puromycin releases mRNA into the cytoplasm and cycloheximide blocks mRNA release from ribosomes, having a positive and negative effect on SGs respectively as confirmed in arsenite-treated cells (Supplementary Fig. 4a, b). Interestingly, the number of M-bodies significantly increased with puromycin and decreased with cycloheximide (Fig. 6a, b), suggesting their involvement in M-phase protein translation regulation and mRNA sequestration. We observed that neither puromycin nor cycloheximide treatment-induced PABP accumulation in M-bodies (Supplementary Fig. 4c).

If M-body formation in M-phase is a solely passive function of mRNA availability, then PKCε would be expected not to impact the effect of puromycin. Quantifying the number of foci upon Blu577 treatment in combination with puromycin (Fig. 6c, d), demonstrated that in fact PKCε inhibition resulted in a global reduction in M-bodies, not only under basal conditions but also in puromycin-treated cells. This indicates that a substantial subset of M-bodies induced by puromycin is under PKCε control and hence PKCε actively contributes to promote M-body formation. Dependency on PKCε activity was substantiated in doxycycline-inducible GFP-PKCεD536N kinase-dead mutant DLD1 cells on knockdown of endogenous PKCε (Supplementary Fig. 4d). We confirmed these findings using the fractionation assay in synchronised DLD1 cells treated with puromycin (Fig. 6e). On treatment with BLU577, both SERBP1 and FMRP levels are reduced in the M-body/SG fraction 3 S normalised to the sum of all bands (band intensity from 12 to 5 and from 50 to 27, respectively). As observed above (Fig. 4d), G3BP is again under PKCε control as its expression is reduced from 15 to 7.7. However, the quantification revealed that LARP4B levels upon translation inhibition are not affected by Blu577 (band intensity from 11 to 13.5).

The formation of M-bodies under conditions of pharmacologically induced mRNA release and the retention of PKCε control, suggests that the concerted action of PKCε to both alter SERBP1-ribosome function and promote M-body formation might impact M-phase translation. To assess this, we employed a ribopuromycylation assay in DLD1 cells synchronised in mitosis. Cells were either treated with DMSO or Blu577 and puromycin in combination with cycloheximide, added 5 min before lysis in order to inhibit re-initiation of ribosomes. As shown in Fig. 6f, PKCε inhibition enhanced active translation as demonstrated by increased puromycylation of proteins. This suggests that PKCε represses protein translation in mitosis, consistent with altered SERBP1 ribosomal association and the promotion of M-body formation.

### SERBP1 is required for successful chromosome segregation and cell division in the PKCε-dependent genome protective pathway

To assess whether SERBP1 contributes to the PKCε-dependent genome protective pathway, we monitored cell division in cells upon SERBP1 knockdown. First, we observed a delay in chromosome alignment on the metaphase plate (Supplementary Fig. 5a). Although this was similar to what was previously observed with PKCε downregulation^23^, the integrity of mitotic spindle structures upon SERBP1 knockdown (Supplementary Fig. 5b) suggests that it may influence this process through an independent pathway.

**Fig. 7:**
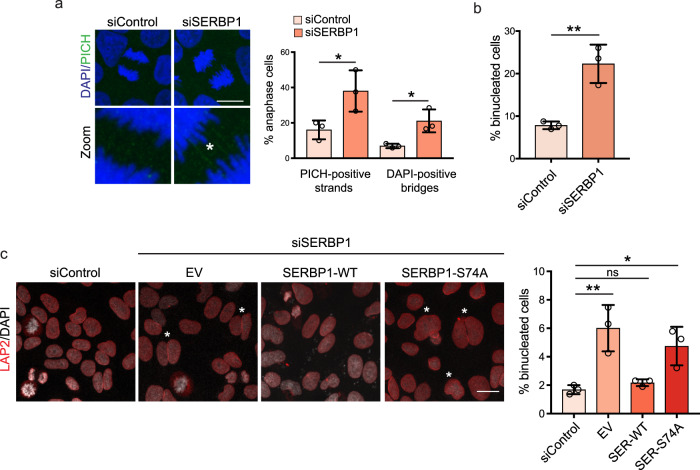
SERBP1 is required for proper chromosome segregation and cell division. **a** Representative images of DLD1 cells in anaphase, transfected with siControl or siSERBP1. Cells were stained with anti-PICH (green) to detect ultra-fine bridges and DAPI (blue). In the zoom, the asterisk indicates a PICH-positive bridge. The percentage of cells with anaphase bridges (PICH or DAPI) is quantified on the right. *n* = 20 cells examined in 3 independent experiments. Error bars, mean ± SD. Unpaired *t* test, *p = 0.04, *p = 0.02. Scale bar, 10 μm. **b** Quantification of binucleated cells transfected with siControl or siSERBP1. *n* = 200 cells examined in 3 independent experiments. Error bars, mean ± SD. Unpaired *t* test, **p = 0.005. (**c**, left) DLD1 cells transfected with siControl and siSERBP1, transiently expressing the empty vector (EV), siRNA resistant FLAG-SERBP1-WT and FLAG-SERBP1-S74A. Cells were stained with anti-LAP2 (red) and DAPI (grey). Scale bar, 10 μm. Asterisks indicate binucleated cells. (c, right) Graph shows the quantification of binucleated cells transfected with siControl and siSERBP1, transiently expressing the empty vector (EV), siRNA resistant FLAG-SERBP1-WT and FLAG-SERBP1-S74A. *n* = 300 cells examined in 3 independent experiments. Error bars, mean ± SD, One way-ANOVA and Tukey’s multiple comparisons test, **p = 0.005, *p < 0.034, ns = 0.94. Source data are provided as a Source Data file.

**Fig. 8:**
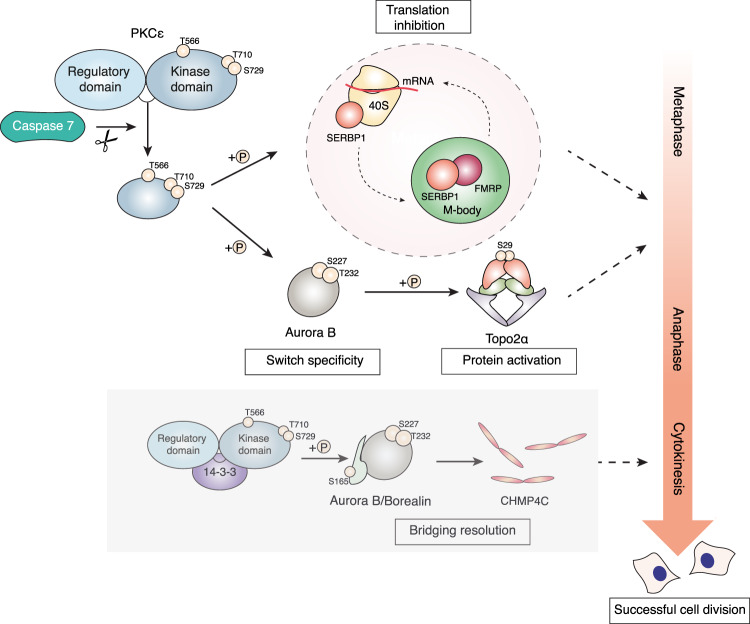
Schematic showing the multiple pathways regulated by PKCε engaged at several steps of mitosis in order to ensure successful cell division. Prior to anaphase, a pool of constitutively active PKCε generated by Caspase 7 acts through Aurora B and Topoisomerase 2α phosphorylation, as previously decribed^4^, or through protein translation repression influencing SERBP1 positioning on the 40S subunit of the ribosome and SERBP1/FMRP M-body formation. Later in cytokinesis, 14-3-3/PKCε complex formation is required for Aurora B phosphorylation and the exit from the abscission checkpoint^48^.

SERBP1 knockdown resulted in multiple phenotypes associated with genomic instability, such as increased PICH-positive ultra-fine bridges, DAPI positive bridges and increased numbers of binucleated cells, which indicate chromosome non-disjunction errors and failed cell division, respectively (Fig. 7a, b). This reveals a role not previously described for SERBP1 in mitosis and it also suggests that SERBP1/FMRP M-bodies may act on the PKCε genome protective pathway. We addressed this by observing cell division in transfected DLD1 cells expressing FLAG-SERBP1 WT or FLAG-SERBP1 S74A (Fig. 7c, d). Although the expression of the WT protein can recover the effect of endogenous SERBP1 knockdown (EV), the S74A mutant displayed an impaired rescue with a significantly higher number of binucleated cells compared to the siControl. Consistent with SERBP1 action, FMRP knockdown, which independently compromises the formation of SERBP1^+^ M-bodies (see above), also resulted in an increased failure to complete cell division (Supplementary Fig. 5c–d). As summarised in Fig. 8, through this novel kinase substrate screening strategy employing the PKCε kinase domain as bait, we have identified SERBP1 as a target impacting mitotic translation and acting in concert with Aurora B to protect cells from division failure (Fig. 8).

## Discussion

In a genome protective pathway, PKCε orchestrates Aurora B function at two specific junctures: at the end of metaphase, the transition to anaphase is delayed to enable chromosome non-disjunction resolution^4,30^ and then again in the later abscission checkpoint^31^, PKCε is activated by 14-3-3^32^ and phosphorylates Aurora B to promote successful mitotic exit^5^. Although Aurora B plays a key role in these PKCε regulated cell cycle delays, we investigated whether its requirement is sufficient or if other PKCε cell cycle targets are involved. Despite the abundance of methods available, it remains challenging to uncover the direct substrates of a protein kinase^33,34^. In vitro^35^ or in cells^36^ currently available approaches have some significant limitations including loss of cellular compartmentalisation, lack of specificity (kinase promiscuity in vitro) and challenges in deconvolution and specific kinase assignment in the context of changing phosphoproteomics profiles in cells/tissues. We addressed this longstanding problem by combining the unambiguity of in vitro approaches with the physiological relevance of in-cell approaches. Based on structural modelling of kinase-substrate interactions we combined genetic incorporation of unnatural, UV-cross-linkable amino acids with in cell photo-crosslinking, an approach widely used for protein-protein interaction studies^9^. A key challenge of this approach is the selection of suitable site(s) for photo-crosslinker insertion. Predictably, certain sites of insertion either destabilise the kinase or impair substrate binding, while other sites are too distant to the site of substrate binding to achieve productive crosslinking. Here, we tested the structural predictions empirically, exploiting the substrate-binding pocket-dependent interaction of the kinase and regulatory domains of the protein, an interaction retained on domain coexpression. This led to the identification of residue E612 as a suitable candidate for sidechain modification.

Among the candidate substrates derived from the crosslinking screen, we identified proteins involved in cell cycle regulation associated with PKCε action, such as PCM-1^37^, required for centrosome separation and function, and Diaph3 involved in actin remodelling^38^. The absence of Aurora B itself in this screen suggests that either this interaction is governed by a distinct binding mode, or that there is an abundance threshold in respect of what is detectable, or perhaps that the Aurora B interaction is more transient than those captured in the screen. Our understanding of the limitations of this screening approach will need to develop from additional studies on PKCε and other kinases. It is interesting to note that a significant number of candidate targets identified in this screen are involved in different aspects of RNA metabolism; this includes SERBP1 which was selected for the purposes of validating the screen and assessing functional relevance.

SERBP1 was validated as a PKCε target in vitro, as demonstrated by the phosphorylation of SERBP1 by recombinant PKCε and the identification of S74 as the dominant phosphorylation site. This phosphorylation was confirmed on co-expression in cells. We described a previously unreported localisation and requirement for SERBP1, which accumulates in granules exclusively in mitosis (M-bodies). RNA degradation induced by RNase treatment leads to M-body dissolution, providing evidence that M-bodies can be defined as ribonucleoprotein structures. PKCε phosphorylation at the S74 site on SERBP1 is decisive for this mitotic event, thus providing a biological validation of the PKCε-SERBP1 relationship uncovered here through the novel substrate screening approach.

Given the role of SERBP1 in mRNA stability in stress granules, we had speculated on the potential relationship between the two structures. M-bodies appear in prometaphase, are dynamically maintained in the cytoplasm until completion of cell division and are not retained in the ensuing G1. On the contrary, stress granules are formed in response to stress in interphase and disassembled when cells enter mitosis^18^. Consistent with this, arsenite treatment results in SERBP1 accumulation in large foci containing PABP in interphase cells and this colocalization is lost when cells enter mitosis. Although the M-bodies are assembled specifically in M-phase, they contain some proteins present in stress granule cores, such as G3BP and FMRP. Bound to untranslated mRNA in the primary aggregation event of SG formation^39^, FMRP interacts with a SERBP1 paralog, Ki-1/57, inducing mRNA translation repression^40^. However, a more recent study demonstrated that the RNA-dependent SERBP1 interaction with FMRP also occurs in the absence of stress, implying that this association may prepare for stress granule formation in case of adverse conditions^17^. The known role of FMRP in mRNA stability^27^, its presence in M-bodies and the mutual dependence on SERBP1, provides a key marker to better understand the role of these structures in mitosis.

Global translation has long been assumed to be considerably reduced in mitosis^41,42^, most likely as a result of the inhibitory hyper-phosphorylation state of translation initiation factors. This dogma has been challenged recently by reports demonstrating that global protein synthesis is not significantly modulated through the cell cycle^43,44^. Here we have observed that protein synthesis in mitosis is suppressed and regulated by PKCε. This is associated with a change in SERBP1 binding to the 40S ribosomal subunit and the accumulation of SERBP1^+^ M-bodies. Even when triggered by the non-specific release of mRNA from ribosomes by puromycin, there remains a PKCε input to M-body formation. The implication of these observartions is that in parallel to the PKCε-Aurora B M-phase pathway recently characterised^4^, there are parallel outputs from PKCε that lead to a decrease in protein translation. Mechanistically, this appears to be effected by a combination of altered interaction between SERBP1 and the 40S ribosomal subunit and the control of M-body formation; drawing from insights into SG action, the latter is likely to contribute to mRNA sequestration and reduced translation. Functionally, the loss of SERBP1 is seen to lead to increased chromosome bridges in anaphase characteristic of premature metaphase/anaphase transition, phenocopying PKCε loss, and ultimately leading to division failures.

## Methods

### Reagents and drugs

All reagents were purchased from Sigma Aldrich unless otherwise stated. Blu577 was kindly provided by Dr Jon Roffey, Cancer Research Technology, UK. DiZASeC was synthesised by X.X. in the laboratory of Prof. Chen Peng^11^ EHNA and Ciliobrevin were obtained from Calbiochem and Cycloheximide from Millipore. All drugs are used at the concentrations stated in the text or figure legends.

### Cell lines, DNA plasmid transfection and siRNA transfection

DLD1, HEK293T and HeLa cell lines were obtained from the Cell Services Science Technology Platform at the Francis Crick Institute. Cells were cultured in DMEM (Thermo Fisher Scientific) with 10% FCS (Thermo Fisher Scientific). DLD1-εWT, DLD1-εD383/451 N and DLD1-εD536N have been engineered from the original cell lines DLD1-FRT-Trex (kindly provided by Prof. Stephen Taylor). Cells were depleted of endogenous protein by siRNA and cultured in DMEM containing 10% FCS and tetracycline (100 ng/ml) for 24 h prior to assay. Cells were treated with inhibitors for the time/s stated. Cell lines were routinely tested for mycoplasma. Cell synchronisation was performed as previously described^4^. Unless otherwise indicated, HEK293T cells were transiently transfected with purified plasmid DNA using the Lipofectamine 2000 reagent (Thermo), for 24–48 h prior to analysis. siRNA transfection was performed using the Lullaby reagent (Oz Biosciences) and the siRNAs listed in Supplementary Table 2 were purchased from Dharmacon and used at 20 nM.

### Genetic incorporation of AbK and photocrosslinking

The site on the protein of interest where AbK is inserted was first mutated to TAG in the expression plasmid followed by co-transfection in a 1:1 ratio with a plasmid (generously provided by the Schultz group^9^) that encodes both an aaRS optimised to recognise AbK, and the tRNA^Pyl^ which recognises the UAG codon. Cell media was replaced with DMEM containing 1 mM AbK prior to the addition of transfection complexes. Transfected cells were incubated and irradiated for 10 min on ice with 365 nm UV light. Cells were then washed briefly with ice-cold PBS and processed for western blot analysis. DiZASeC was similarly incorporated, using a working concentration of 200 μM, and where the AbKRS construct was adapted for optimal incorporation efficiency by making M274A and A313S mutations (to give the PylRS sequence used previously) and appending a NES sequence at the N-terminus to increase cytoplasmic localisation.

### Protein purification

Purification of recombinant PKCε, both full-length and catalytic domain, was carried out by the Structural Biology STP of the Francis Crick Institute. GST-tagged PKCε catalytic domain was cloned into a pFastBacDual vector also containing the full-length human PDK1 cDNA. Sf21 cells were transfected with bacmids generated using the DH10Bac *E. coli* strain. The initial viral stock was amplified to approximately 1 × 10^8^ pfu/ml through passaging in Sf21 cells. For protein expression, 500 ml of Sf21 cells at 1 × 10^6^ cells/ml were infected at MOI:2 with the baculovirus co-expressing PKCε and PDK1. The cells were harvested 72 h postinfection and re-suspended in 50 ml of 25 mM Tris pH 8.0, 150 mM NaCl, 1 mM EDTA, 1 mM DTT, 1 mM ATP, with protease inhibitors (tablet, Roche). The cell suspension was sonicated and passed through a 20 gauge needle several times. Insoluble material was pelleted by 45000 x *g* centrifugation. The soluble fraction was automatically loaded onto a GST 5 ml trap column using a sample pump. The eluted fraction was added to a 50 ml Falcon containing 10 ml of buffer, GST-3C protease and a Roche tablet to avoid unwanted proteolytic break-down. Around 10 h after digestion, the mixture was loaded onto a Resource15Q column. The buffers passed through the column were as follows. Low salt: 25 mM Hepes pH 7.6, 25 mM NaCl, 1 mM DTT, 1 mM EDTA, and high salt: 25 mM Hepes pH 7.6, 500 mM NaCl, 1 mM DTT, 1 mM EDTA. A subset of the different fractions was analyzed by TapeStation to identify fractions containing PKCε. The fractions F8-F11 were pooled and concentrated. The final volume was 300 µl with a concentration of 0.390 mg/ml using the Nanodrop and an extinction coefficient of 38.000 M^−1^ cm^−1^ and a MW of 39 kDa. A final concentration of 10% (v/v) glycerol was added to the concentrated protein and the protein was frozen at −20C.

### Co-immunoprecipitation and immunoblotting

For immunoprecipitation, cells were lysed in 1% Triton X-100 lysis buffer (50 mM Tris pH 7.5, 150 mM NaCl, 1% Triton-X, 10 mM NaF) supplemented with PhosStop and Complete EDTA-free Protease Inhibitor Cocktail (Roche). Following 10 min incubation on ice, lysates were cleared through centrifugation at 20000 × *g* at 4 °C for 10 minutes. 2x LDS sample buffer (Invitrogen) 100 mM DTT was added, samples were boiled and loaded to either NuSep Tris-HEPES or NuPage Bis-Tris (Thermo) polyacrylamide gels, and separated by SDS-PAGE and transferred to PVDF membranes. In the case of FLAG-tag immunoprecipitations, FLAG M2 magnetic beads (Sigma) with antibody pre-conjugated to beads were first washed and subsequently added to lysates. In the case of other immunoprecipitations, antibodies were bound to Protein G Dynabeads magnetic beads (Thermo) in 0.02% Tween 20/PBS at room temperature for 10 minutes. For immunoblotting, cells were lysed in 1X NuPAGE LDS sample buffer (Thermo) and sonicated for 3 × 10 s on ice. Proteins were separated by SDS–PAGE and transferred to PVDF membranes (Millipore). Membranes were blocked for 1 hour at room temperature in PBS/0.01% Tween and 3% BSA for the following antibodies: rabbit anti-PKCε (Santa Cruz (sc-214), 1:1000), rabbit anti-PKCε pT566 (In house, 1:1000), rabbit anti-PKCe pT710 (In house, 1:1000), rabbit anti-PKCε pS729 (Invitrogen (44-977 G), 1:1000), rabbit anti-PKCε pSer substrate (Cell Signalling Technology (2261), 1:1000), mouse anti-GAPDH (Millipore (MAB374), 1:5000), mouse anti-Myc (Cell Signalling Technology (2276), 1:2000), mouse anti-FLAG M2 (Sigma (B3111), 1:2000), rabbit anti-SERBP1 (Abcam (55993), 1:1000), mouse anti-α-tubulin (in house, 1:1000), mouse anti-PABP (Santa Cruz (sc-166027), 1:1000), mouse anti-FMRP (Millipore (MAB2160), 1:1000), rabbit anti-LARP4B (Novus Biologicals (NBP1-80890), 1:1000), mouse anti-Puromycin (Millipore (MABE341), 1:3000), rabbit anti-EGFR (Cell Signaling (4267), 1:1000), mouse anti-GM130 (BD Biosciences, (610823), 1:1000), mouse anti-G3BP (BD Biosciences, (611126) 1:1000), mouse anti-RPS6 (Cell Signaling (2217), 1:1000), mouse anti-phospho Ser/Thr-pro MPM2 (Millipore (05368), 1:2000), anti-Vimentin (Santa Cruz (sc-6260), 1:1000), rabbit anti-TopoIIα phosphoSer29 (made in house, 1:1000), mouse anti-TopoIIα (Millipore (MAB4197), 1:2000), rabbit anti-Aurora B phosphoSer227 (made in-house, 1:500). Antibodies were subsequently detected using HRP-conjugated mouse or rabbit secondary antibody (GE Healthcare (NA931 or NA934), 1:2000) and Luminata Classico ECL reagent (Millipore) and imaged using an ImageQuant LAS 4000 (GE) set to detect chemiluminescence. For visualisation of phosphorylation, membranes at this stage were treated with the ProQ diamond blot stain kit (Thermo) according to the manufacturer’s instructions and imaged using ImageQuant LAS 4000 (GE) set to detect fluorescence. Band densitometry was carried out using ImageJ software and normalised to a loading control.

### Peptide array

Peptide arrays were synthesised by the Peptide Chemistry STP of the Francis Crick Institute using a Multipep Peptide Synthesiser (Invatis). The arrays were comprised of peptides of 20 amino acids in length attached to a cellulose sheet, with the C-termini of peptides anchored to the array, and the acetylated N-terminus of the peptide extending out from the array. Peptides corresponded to the entire sequence of SERBP1, with a one amino acid shift along the SERBP1 sequence with each consecutive peptide. The phosphorylation assay was carried out by incubating the membrane, in a partly sealed sachet, with agitation for 5 minutes at room temperature with 4 ml of kinase buffer composed of 4 μg/ml PKCε catalytic domain, 10 mM MgCl_2_, 0.1 mg/ml BSA, and 100 μM ATP (at 62.5 μCi/ml). The membrane was then washed three times with 30% acetic acid, three times with dH_2_O, and three times with 0.1 M NaOH for 5 min each. Following this, peptide phosphorylation was visualised through the exposure of the membrane to X-ray film.

### HaloTag purification for mass spectrometry

HEK293T cells were cultured in a 15 cm dish. Site-selective incorporation of AbK or DiZASeC into PKCε and subsequent photocrosslinking was carried out as described above. Cells were first scraped into 1 ml ice-cold PBS, and pelleted by centrifuging at 500 × *g* for 5 min using a benchtop centrifuge. Cells were lysed using 300 μl of HaloTag lysis buffer, 10 min at room temperature. The lysate was then cleared through centrifugation at 20000 × *g* for 15 min at 4 °C, supernatant transferred to a new tube and diluted to 1 ml with 700 μl 1x TBS. A 50 μl aliquot of this supernatant was taken into 2x LDS sample buffer and boiled for SDS-PAGE analysis as the input to the purification. If necessary, protein concentration at this point was determined using a Microplate BCA assay kit (Thermo) to normalise the amount of protein loaded onto the HaloLink resin in the next step. 200 μl of HaloLink resin slurry per sample was equilibrated through washing 4 times with 1 ml of TBS containing 0.05% Triton X-100. The lysate was the added to the washed HaloLink resin, and suspension shaken for 1 h at 1,200 rpm at room temperature using a ThermoMixer. The beads were then pelleted by centrifuging at 800 × *g* for 2 min using a benchtop centrifuge, and 50 μl of the supernatant taken into 2x LDS sample buffer to assess binding of PKCε to beads through comparison by western blot with the amount present in the lysate. Beads were then washed with 1% SDS in PBS (pH 8.0) three times, before being washed with 7 M Urea, 0.5 M NaCl, 1% Triton X-100, in Tris (pH 8.0) twice. The beads were then washed three more times with TBS containing 0.05% Triton X-100. If DiZASeC cleavage was not required, beads were directly submitted to on-bead trypsinolysis and mass spectrometry analysis. If DiZASeC cleavage was required, at this stage beads were resuspended in 100 μl of 1% SDS in PBS (pH 8.0), and H_2_O_2_ added to 7 mM. This suspension was then rotary agitated at 1,200 rpm at room temperature using a ThermoMixer for 2 h. The supernatant was the subjected to SP3 purification for mass spectrometry analysis, and the beads were submitted for on-bead trypsinolysis treatment.

### Immunofluorescence microscopy and image analysis

Cells were grown on 13 mm glass coverslips and fixed and permeabilized with PHEM buffer (60 mM PIPES pH6.8, 25 mM HEPES pH7.4, 10 mM EGTA pH8, 4 mM MgSO_4_, 4% paraformaldeyhyde and 0.1% Triton X-100) for 20 min. Cells were then incubated in blocking buffer (3% BSA/PBS-0.1%Tween) and probed using the following primary antibodies: rabbit anti-SERBP1 (Abcam (55993), 1:300), mouse anti-FMRP (Merk Millipore (MAB2160), 1:300), mouse anti-PABP (Santa Cruz (sc-166027), 1:300), mouse anti-αtubulin (in house, 1:1000), rat anti-Ago2 (Sigma-Aldrich (HPA058075), 1:300), mouse anti-RPS6 (Cell Signalling (2217), 1:300), rabbit anti-PICH (Abnova (H00054821-D01), 1:300), mouse anti-LAP2 (BD Biosciences (611000), 1:300), rabbit anti-LARP4B (Novus Biologicals (NBP-80890), 1:300). The secondary antibodies used were obtained from Thermo Fisher Scientific and were all used diluted in blocking buffer 1:1000: goat anti-rabbit Alexa Fluor 488 (A11008), goat anti-mouse Alexa Fluor 555 (A21422), goat anti-rabbit Alexa Fluor 555 (A21428), goat anti-mouse 488 (A11001). All coverslips were mounted using ProLong Gold Diamond with DAPI (Invitrogen). All the images were acquired using an inverted laser scanning confocal microscope (Carl Zeiss LSM 780) equipped with a 40x and a 63x Plan-APOCHROMAT DIC oil-immersion objective. Image analysis was carried out using the ZEN analysis software. All images are shown as maximum intensity projections. For quantification of the mean number of SERBP1 foci per mitotic cell, all immunofluorescence images were gamma corrected in FIJI and quantified by a custom-built script using the commercial software package MATLAB (MATLAB R2017b, MathWorks). To detect SERBP1 foci, a percentile-based intensity filter was applied to all control images in each experiment and the average intensity value was calculated. Subsequently, this intensity value was used to threshold all other images and conditions. At least 15 images per condition, unless otherwise stated, were analysed and the Mean and S.E.M. of at least three experiments was quantified.

### Live microscopy

Cells were cultured on LabTek chambered coverglass slides (Nunc) in Leibovitz CO2-independent media (Thermo Fisher Scientific). A low light level inverted microscopy (Nikon TE2000) imaging system equipped with a laminar-flow heater to maintain a constant temperature of 37 ± 0.001 °C, a PlanFluor 40’ DIC lens and a Xenon lamp for fluorescent excitation. Images were taken using a high quantum efficiency CCD camera (Andor Ixon) every 5 min using MetaMorph (Version 6.3) software.

### Fractionation assay

Pellets from cells grown in 15 cm dishes were flash frozen in liquid nitrogen and held for at least 1 h at −80 °C. Samples were thawed 5 minutes on ice and resuspended in 1 ml lysis buffer containing 50 mM Tris-HCl pH 7.5, 100 mM Potassium Acetate, 2 mM Magnesium Acetate, 0.5 mM DTT, 0.5% NP40, 1:5000 antifoam B, 1 EDTA protease inhibitor, and lysed by pipetting 5X through a 25 G needle on ice. Samples were centrifuged 5 minutes at 1000 × *g* at 4 °C and the supernatant was spun again at 17,000 x *g* at 4 °C for 25 min. The resulting supernatant was used as input for cytoplasmic material (2 S) and the pellet was resuspended in 300 µL lysis buffer and centrifuged at 800 x *g* at 4 °C for 2 min to finally obtain a third pellet (3 P) and a third supernatant (3 S).

### RNase A treatment and Ribopuromycylation assay

DLD1 cells were treated for 1 or 5 min with 0.1% Triton at room temperature. After washes with PBS, 100 µg/mL RNase A was added for 10 minutes on ice. Cells were fixed with 4% paraformaldeyhyde for 15 min at room temperature, washed with PBS and stained for immunofluorescence microscopy as described. In the translation activity assays, cells were pre-treated with 20 μg/mL Puromycin (Sigma-Aldrich) or 10 μg/mL Cycloheximide (Millipore) for 1 h to inhibit ribosomal translational or 50 μg/mL Puromycin with 100 μg/mL Cycloheximide for 5 min to label active translation.

### Structural models and iCLIP methods

Structural models were generated using Pymol. iCLIP was performed as in Blazquez et al.^45^, with the exception that 10nt UMIs were used. Mitotic samples and Blu577 treatment were performed as described above, and samples were prepared in triplicate. The data were aligned using STAR^46^. Reads were first aligned to a genome containing the ribosomal DNA repeat, and all snRNA, tRNA and snoRNA sequences from the GENCODE 29 annotation with multimapping permitted, and unmapped reads were then aligned to the genome with only unique reads being retained. For analysis of rRNA crosslinking, crosslink counts were normalised by the total number of rRNA crosslinks for that sample. Metaprofile crosslinks were smoothed using a gaussian kernel of width 9 and standard deviation of 2.

### Statistical analysis

For experiments where the data includes more than two conditions, a one-way ANOVA using multiple comparisons was used, in all other cases an unpaired t-test was used for analysis. Prism software (Graphpad) was used for all calculations. When exact p values are not included in the figure legend, the level of statistical significance is represented as follows: ns = *P* > 0.05, *P ≤ 0.05, **P ≤ 0.01, ***P ≤ 0.001 and ****P ≤ 0.0001. Statistical analysis of iCLIP data was performed using R.

### Statistics and reproducibility

Immunofluorescence analysis to detect SERBP1 M-bodies as in Fig. 3a, b and Fig. 4c has been performed in at least 20 independent experiments. Experiments in Fig. 1b, 1c, Supplementary Fig. 1c, Supplementary Fig. 2e–g, Supplementary Fig. 5b–c were repeated at least three times with similar results. The experiments shown in Fig. 4a, c, d, Fig. 6a for the FMRP staining, Fig. 6c, e, Supplementary Fig. 1b and d, Supplementary Fig. 3b, Supplementary Fig. 3g were independently repeated twice with similar results. Experiments in Fig. 4e, Supplementary Fig. 1e, f, Supplementary Fig. 2c, d, Supplementary Fig. 3a and c, Supplementary Fig. 3e, f, Supplementary Fig. 4a–d, Supplementary Fig. 5a were performed once.

### Reporting summary

Further information on research design is available in the Nature Research Reporting Summary linked to this article.

## Supplementary information

Supplementary Information

Description of Additional Supplementary Files

Supplementary Movie 1

Reporting Summary

## Data Availability

Source data are provided with this paper. The iCLIP data generated in this study have been deposited in the ArrayExpress database under accession code E-MTAB-10830. The mass spectrometry proteomics data have been deposited to the ProteomeXchange Consortium via the PRIDE^47^ partner repository with the dataset identifier PXD029091. Source data are provided with this paper.

## Source data

Source Data
