# Supplementary material for: A genetically-encoded crosslinker screen identifies SERBP1 as a PKCε substrate influencing translation and cell division
Source: Nat Commun. 2021 Nov 26;12:6934. doi: 10.1038/s41467-021-27189-5 (PMC8626422; doi:10.1038/s41467-021-27189-5)
Supplement: Supplementary file 1 — Supplementary Information [file 41467_2021_27189_MOESM1_ESM.pdf]

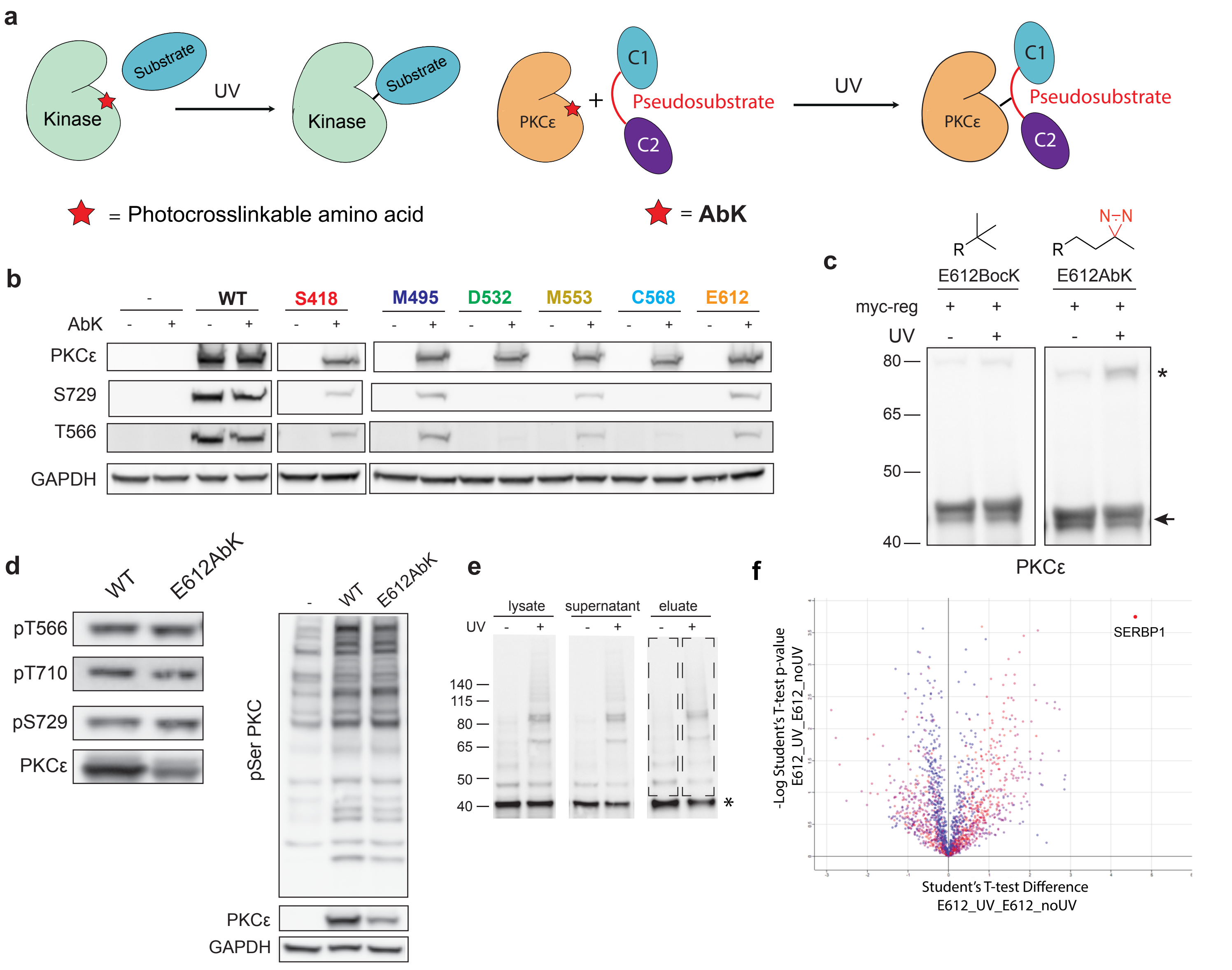

Supplementary Figure 1. The incorporation of the UV-photocrosslinkable un-natural amino acid (Abk) in the E612 site of PKCε resulted as a valuable tool to identify SERBP1 as novel substrate of PKCε. (a) Scheme representing the approach using AbK for crosslinking a kinase to its substrates upon UV irradiation (left) and the assay used to assess sites of AbK incorporation into the PKC catalytic domain (right). (b) Full-length PKCε WT or TAG mutants were expressed in HEK-293T cells either with or without AbK added to the media. Lysates were assessed for both PKCε expression and PKC priming phosphorylations on two sites, T566 and S729. GAPDH is used as loading control. (c) PKCε E612TAG catalytic domain and PKCε regulatory domain expressed in HEK-293T cells under AbK (AbKRS and 1 mM AbK), or BocK (PylRS and 1 mM BocK) incorporation conditions. Cells were irradiated with 365 nm UV light, subsequently lysed and analysed by western blot using a PKCε antibody. The arrow indicates the free PKCε catalytic domain and the asterisk the UV-induced crosslinked species. (d) PKCεWT or E612TAG catalytic domain were expressed in HEK-293T cells under AbK incorporation conditions. Lysates were analysed by western blot using an antibody recognising anti-PKCε(pT566), anti-PKCε(pT710), anti-PKCε(pS729) (left-hand panel) and using an antibody recognising phosphorylated peptides conforming to the PKC consensus sequence (right-hand panel). Anti-PKCε antibody has been used to assess total PKCε expression and GAPDH was employed as a loading control. (e) HEK-293T cells expressing PKCε E612TAG under AbK incorporation conditions, exposed or not to 365 nm UV light. In the eluted samples, bands from the gel above the molecular weight of PKCε catalytic domain (> 40 kDa) were excised (see black boxes) from both UV positive and negative conditions and submitted to mass spectrometry analysis. Label-free quantification (LFQ) was used to compare protein abundance between the positive and negative conditions. (f) Volcano plot showing the results from the mass spectrometry experiment. Unpaired t-test, SERBP1 is highlighted in the plot as a significant hit. Proteins detected with high peptide intensities are shown in blue, and those identified with low intensities shown in magenta. (a) Scheme representing the approach using AbK for crosslinking a kinase to its substrates upon UV irradiation (left) and the assay used to assess sites of AbK incorporation into the PKC catalytic domain (right). (b) Full-length PKCε WT or TAG mutants were expressed in HEK-293T cells either with or without AbK added to the media. Lysates were assessed for both PKCε expression and PKC priming phosphorylations on two sites, T566 and S729. GAPDH is used as loading control. (c) PKCε E612TAG catalytic domain and PKCε regulatory domain expressed in HEK-293T cells under AbK (AbKRS and 1 mM AbK), or BocK (PylRS and 1 mM BocK) incorporation conditions. Cells were irradiated with 365 nm UV light, subsequently lysed and analysed by western blot using a PKCε antibody. The arrow indicates the free PKCε catalytic domain and the asterisk the UV-induced crosslinked species. (d) PKCεWT or E612TAG catalytic domain were expressed in HEK-293T cells under AbK incorporation conditions. Lysates were analysed by western blot using an antibody recognising anti-PKCε(pT566), anti-PKCε(pT710), anti-PKCε(pS729) (left-hand panel) and using an antibody recognising phosphorylated peptides conforming to the PKC consensus sequence (right-hand panel). Anti-PKCε antibody has been used to assess total PKCε expression and GAPDH was employed as a loading control. (e) HEK-293T cells expressing PKCε E612TAG under AbK incorporation conditions, exposed or not to 365 nm UV light. In the eluted samples, bands from the gel above the molecular weight of PKCε catalytic domain (> 40 kDa) were excised (see black boxes) from both UV positive and negative conditions and submitted to mass spectrometry analysis. Label-free quantification (LFQ) was used to compare protein abundance between the positive and negative conditions. (f) Volcano plot showing the results from the mass spectrometry experiment. Unpaired t-test, SERBP1 is highlighted in the plot as a significant hit. Proteins detected with high peptide intensities are shown in blue, and those identified with low intensities shown in magenta.

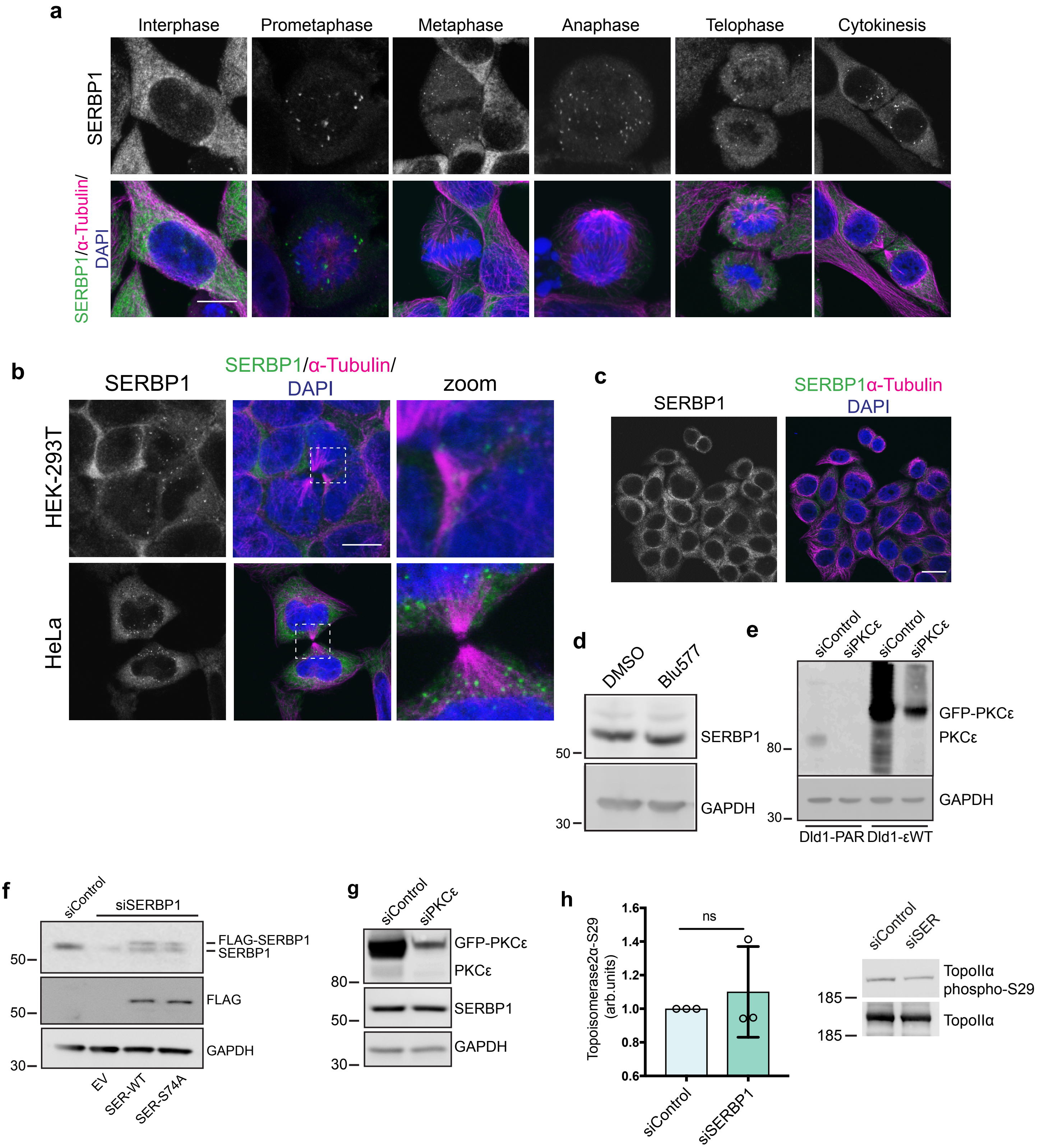

Supplementary Figure 2. SERBP1 M-bodies are exclusively expressed in mitotic cells in several tumour-derived cell lines. (a-c) SERBP1 is labelled in green, α-Tubulin in magenta and DAPI in blue. Scale bar, 10μm. (a) SERBP1 is diffuse in the cytoplasm in interphase and enriched in M-bodies from prometaphase to cytokinesis. (b) Images illustrating SERBP1 accumulation in M-bodies in HEK-293T and HeLa cells. Zoom panels show SERBP1 M-bodies in proximity of the cleavage furrow in mitotic cells. (c) DLD1 cells were synchronised in mitosis and, following mitotic shake off, replated to observe M-body presence in the new interphase. Scale bar, 10μm. (d-g) GAPDH is used as a housekeeping control. (d) Western blot analysis of endogenous SERBP1 in cells treated with DMSO or 500nM Blu577 for 1 hour. (e) Western blot analysis of endogenous PKCε and GFP-PKCε levels in DLD1-PAR and DLD1ε-WT cells transfected with siControl and siPKCε. (f) Western blot analysis of DLD1 cells transfected with siControl or siSERBP1 for 48 hours and transiently transfected with an empty vector (EV), FLAG-SERBP1-WT and FLAG-SERBP1-S74A mutant. Blots were stained with SERBP1, FLAG and GAPDH antibodies. (g) Western blot of siControl and siPKCε-transfected DLD1-εD383/451N cells. Samples were immunoblotted for PKCε and SERBP1. Exogenous PKCε is indicated as GFP-PKCε. (h) Quantification of Topoisomerase 2α phospho-S29 normalized on total Topoisomerase 2α in Triton-X 100 extracts from cells transfected with siControl and siSERBP1. siSERBP1 values were normalised on siControl. Error bars, mean ± SD of n=3 independent experiment, ns=0.58.

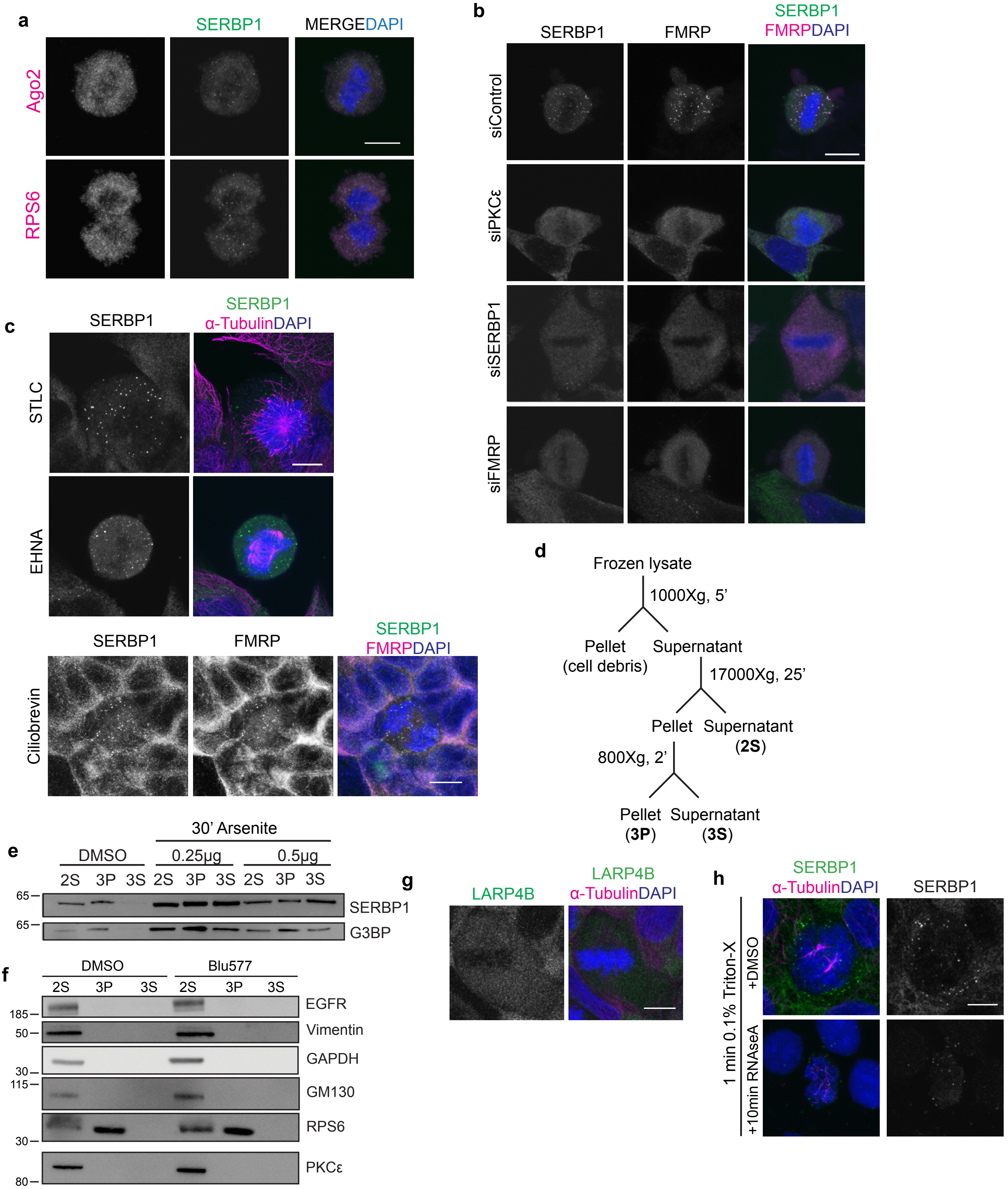

Supplementary Figure 3. Characterization of the SERBP1/FMRP M-bodies using immunofluorescence and a fractionation assay. (a) DLD1 cells labelled with SERBP1 (green), Ago2 or RPS6 (magenta) and DAPI (blue). Scale bar, 10µm. (b) Representative images of cells transfected with siControl, siPKCε, siSERBP1 or siFMRP. SERBP1 is labelled in green, FMRP in magenta and DAPI in blue. Scale bar, 10mm. (c) DLD1 mitotic cells treated with 1µM S-Trityl-L-cysteine (STLC), 100µM EHNA or 20µM ciliobrevin. Cells were labelled with anti-SERBP1 (green), anti-αTubulin or anti-FMRP (magenta) and DAPI (blue). Scale bar, 10µm. (d) Description of the fraction assay used to obtain 2S (second supernatant), 3P (third pellet) and 3S (third supernatant) fractions. (e) Log-phase DLD1 cells treated with DMSO, 0.25µg or 0.5µg arsenite for 30 minutes were fractionated and run on SDS-gels. Samples were immunoblotted for SERBP1 and G3BP. (f) Western blot using DLD1 cells obtained from the fractionation assay, 1 hour treated with DMSO or 500nM Blu577. Samples were immunoblotted for the indicated proteins. (g) Representative image of a DLD1 cell in metaphase, fixed and stained for LARP4B (green), α-Tubulin (magenta) and DAPI (blue). Scale bar, 10µm. (h) Cells treated with 0.1% Triton-X 100 for 1 minute. SERBP1 in green, α-Tubulin in magenta and DAPI in blue. Scale bar, 10µm.

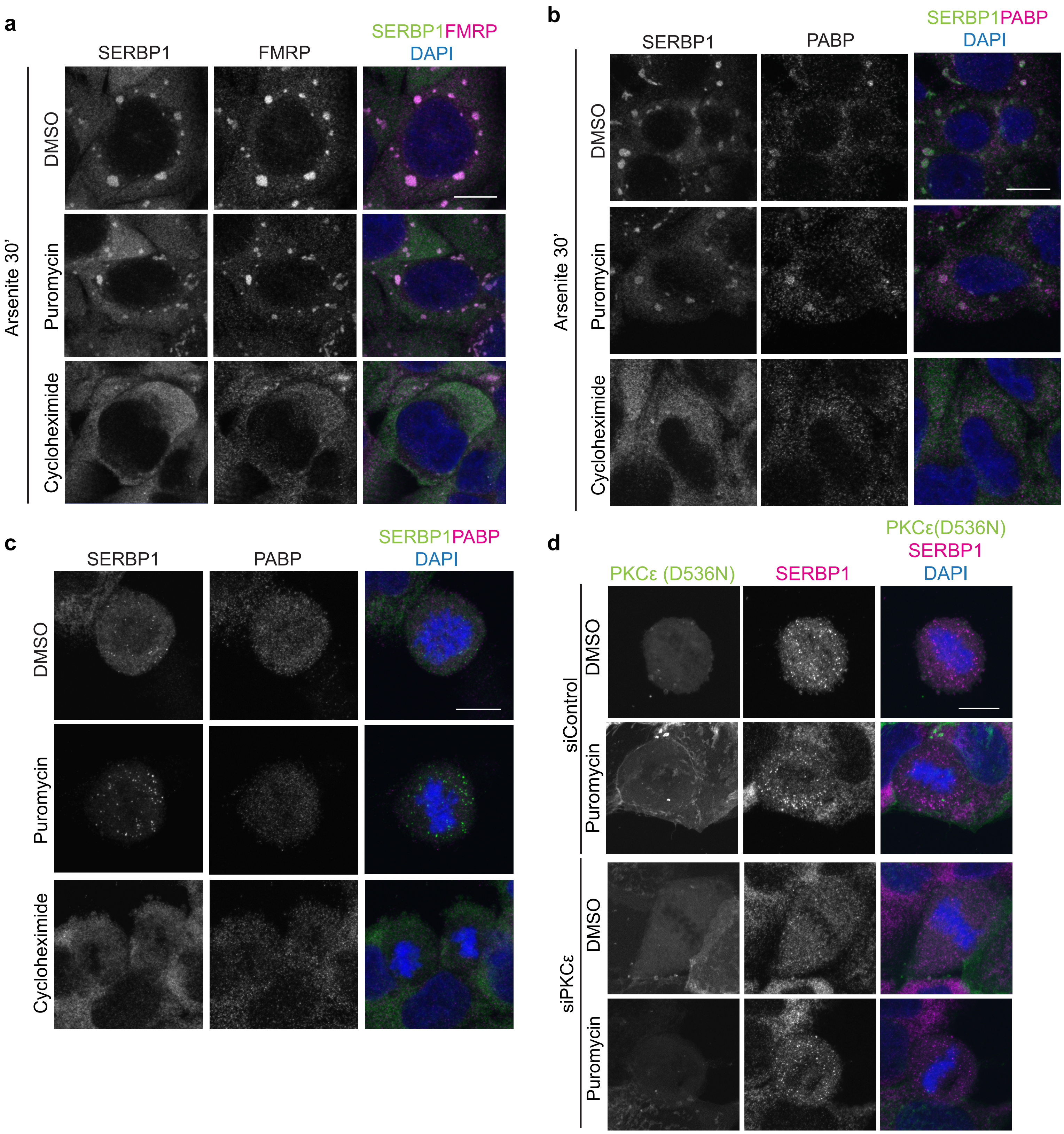

Supplementary Figure 4. Assessment of the translation inhibitors Cycloheximide and Puromycin on stress granules formation. (a,b) DLD1 cells were treated for 30 minutes with arsenite and 5 minutes with DMSO, or 20 μg/ml puromycin or 10 μg/ml cycloheximide as indicated. Cells were labelled with antisera to: SERBP1 (green), FMRP (magenta, a) or PABP (magenta, b) and DAPI (blue). Scale bar, 10μm. (c) DLD1 cells were treated for 5 minutes with DMSO, puromycin or cycloheximide, then fixed and stained for: SERBP1 (green), PABP (magenta) and DAPI (blue). Scale bar, 10μm. (d) Doxycycline-inducible GFP-PKCεD536N kinase dead cells were transfected with siControl or siPKCε and treated for 1 hour with DMSO or 20 μg/ml Puromycin. Cells were imaged for SERBP1 (magenta) and DAPI (blue). GFP-PKCε is detected in green. Scale bar, 10μm.

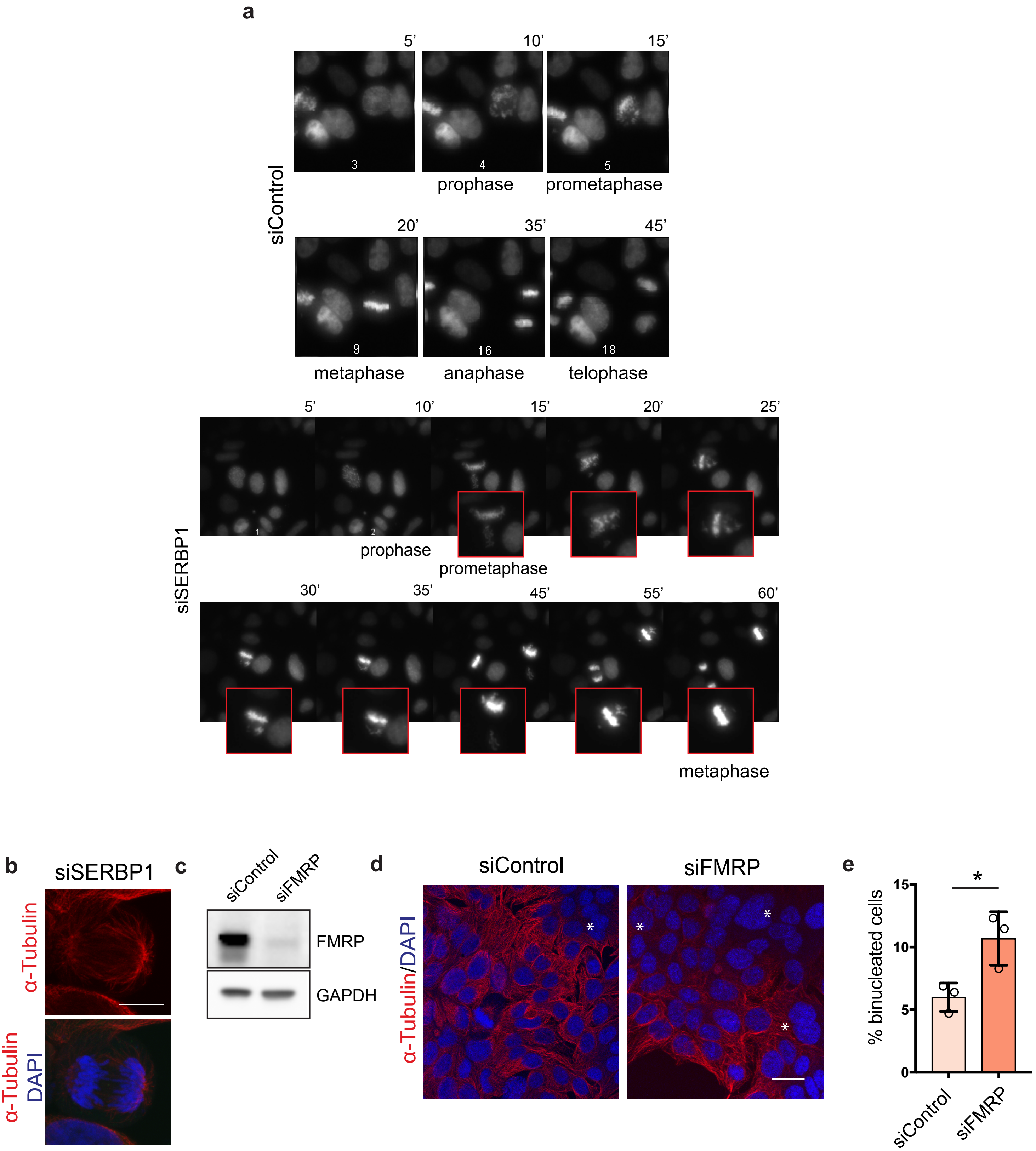

Supplementary Figure 5. Effect of SERBP1 or FMRP downregulation on chromosome alignment in metaphase or cell division. (a) HeLa H2B-mCherry cells transfected with siControl (top) and siSERBP1 (bottom) and imaged every 5 minutes. Mitotic phases are indicated. Zoom shows a cell progressing from prometaphase to metaphase. (b) DLD1 cell in anaphase. Mitotic spindle is shown with α-Tubulin (red) staining and DNA with DAPI (blue). Scale bar, 10μm. (c) Western blot representing DLD1 transfected with siControl and siFMRP. Samples were immunoblotted for FMRP and GAPDH was used as housekeeping. (d) DLD1 cells transfected with siControl or siFMRP and stained with α-Tubulin (red) and DAPI (blue). Asterisks indicate binucleated cells. Scale bar, 10μm. (e) Quantification of binucleated cells (as percentage) in cells transfected with siControl or siFMRP. n=300 cells examined over 3 independent experiments. Error bars, mean ± SD. Unpaired t-test, *p=0.03.

Gene Name	Protein	Score	RNA	Cell cycle/Mitosis	Candidate PKCe sites	PHOSPHOSITE
HNRNPL	Heterogeneous nuclear ribonucleoprotein L	-6.88	Y		S357, T487, S531	T487, S531
SLC39A7	Zinc transporter SLC39A7	-6.43			S293	S293
COQ5	2-methoxy-6-polyprenyl-1,4-benzoquinol methylase	-5.30				
DIAPH3	Protein diaphanous homolog 3	-5.28		Y	S149,S851, S1091	S149, S1091
ACLY	ATP-citrate synthase	-4.95			T278, S839, S979	S839, S979
Q08ES8	60S ribosomal protein L11	-4.45	Y		S58	
SNRNP70	U1 small nuclear ribonucleoprotein 70 kDa	-3.63	Y			
NKAP;NKAPL	NF-kappa-B-activating protein;NKAP-like protein	-3.50	Y		S400	
PCM1	Pericentriolar material 1 protein	-3.19		Y	S220, S1318	S1318
EDC4	Enhancer of mRNA-decapping protein 4	-2.96	Y		S1389	S1389
DDX20	Probable ATP-dependent RNA helicase DDX20	-2.57	Y		T482, S743	S743
CCAR2	Cell cycle and apoptosis regulator protein 2	-2.10	Y	Y	S249, T421, S534	
LARP4B	La-related protein 4B	-2.00	Y		S498	S498
NISCH	Nischarin	-1.49		Y	S83, S940, S1484	
CACYBP	Calcyclin-binding protein	-1.16				
HSPH1	Heat shock protein 105 kDa	-1.13				
HSP90AA1	Heat shock protein HSP 90-alpha	-1.03	Y	Y	T88, S211	T88, S211
SERBP1	Plasminogen activator inhibitor 1 RNA-binding protein	-0.79	Y		S74	S74
CSNK1E	Casein kinase I isoform epsilon	-0.62	Y	Y	S377	S377

Supplementary Table 1. List of the potential PKCε substrates identified employing the incorporation of the photocrosslinkable un-natural amino acid DiZASeC. Hits listed are sorted based on their Welch Score. The table indicates whether the hits are known to bind RNA, to be involved in the regulation of the cell cycle or mitosis and what are the potential phosphorylation sites based on the PKC consensus sequence or as described on Phosphosite Plus.

Scrambled control pool D-001206-13-20	5’-UAGCGAUAAACACAUCAA-3’<br>5’-UAAGGCUAUGAAGAGAUAC-3’<br>5’-AUGUAUUGGCCUGUAUUAG-3’<br>5’-AUGAACGUGAAUUGCUCAA-3’
SERBP1 si3 J-020528-11	5’-GGGUGAAGGAGGCGAAUUU-3’
PKCε si1 D-004653-01	5’-GGGCAAAGAUGAAGUAUAU-3’
FMR1 si2 J-019631-06	5’-GAUGAUAAAGGGUGAGUUU-3’

Supplementary Table 2. Sequences of the siRNAs used in the study.
