# Supplementary material for: A genetically-encoded crosslinker screen identifies SERBP1 as a PKCε substrate influencing translation and cell division
Source: Nat Commun. 2021 Nov 26;12:6934. doi: 10.1038/s41467-021-27189-5 (PMC8626422; doi:10.1038/s41467-021-27189-5)
Supplement: Supplementary file 2 — Description of Additional Supplementary Files [file 41467_2021_27189_MOESM2_ESM.pdf]

Description of Additional Supplementary Files

File Name: Supplementary Movie 1

Description: SERBP1 M-bodies in a metaphase cell. Video rendering (maximum intensity projection) of the same cropped volume in Figure 3a; grid spacing: 2 µm. Images and video have been obtained with the software Imaris Bitplane 9.2.1. SERBP1 is coloured in green, α-Tubulin in magenta and DNA in blue.
