# Supplementary material for: A genetically-encoded crosslinker screen identifies SERBP1 as a PKCε substrate influencing translation and cell division
Source: Nat Commun. 2021 Nov 26;12:6934. doi: 10.1038/s41467-021-27189-5 (PMC8626422; doi:10.1038/s41467-021-27189-5)
Supplement: Supplementary file 4 — Reporting Summary [file 41467_2021_27189_MOESM4_ESM.pdf]

nature research

Corresponding author(s): Peter Parker

Last updated by author(s): Oct 13, 2021

Reporting Summary

Nature Research wishes to improve the reproducibility of the work that we publish. This form provides structure for consistency and transparency in reporting. For further information on Nature Research policies, see our Editorial Policies and the Editorial Policy Checklist.

Statistics

For all statistical analyses, confirm that the following items are present in the figure legend, table legend, main text, or Methods section.

n/a	Confirmed	
☐	☒	The exact sample size (n) for each experimental group/condition, given as a discrete number and unit of measurement
☐	☒	A statement on whether measurements were taken from distinct samples or whether the same sample was measured repeatedly
☐	☒	The statistical test(s) used AND whether they are one- or two-sided<br>*Only common tests should be described solely by name; describe more complex techniques in the Methods section.*
☐	☒	A description of all covariates tested
☐	☒	A description of any assumptions or corrections, such as tests of normality and adjustment for multiple comparisons
☐	☒	A full description of the statistical parameters including central tendency (e.g. means) or other basic estimates (e.g. regression coefficient) AND variation (e.g. standard deviation) or associated estimates of uncertainty (e.g. confidence intervals)
☐	☒	For null hypothesis testing, the test statistic (e.g. F, t, r) with confidence intervals, effect sizes, degrees of freedom and P value noted<br>*Give P values as exact values whenever suitable.*
☒	☐	For Bayesian analysis, information on the choice of priors and Markov chain Monte Carlo settings
☒	☐	For hierarchical and complex designs, identification of the appropriate level for tests and full reporting of outcomes
☒	☐	Estimates of effect sizes (e.g. Cohen's d, Pearson's r), indicating how they were calculated

Our web collection on statistics for biologists contains articles on many of the points above.

Software and code

Policy information about availability of computer code

Data collection	Image-J 1.50i analysis software<br>Fiji (Version 2.0.0-rc-69/1.52p)<br>Zen (Version 2.3 SP1)<br>ImageQuant Las 4000 (Version 1.1)<br>custom-built script in MATLAB (MATLAB R2017b)<br>STAR 2.7.9a<br>Pymol2.5
Data analysis	Prism software - Graphpad (version 7.0c); Statistical analysis of iCLIP data was performed using R (version 4.0.5)

For manuscripts utilizing custom algorithms or software that are central to the research but not yet described in published literature, software must be made available to editors and reviewers. We strongly encourage code deposition in a community repository (e.g. GitHub). See the Nature Research guidelines for submitting code & software for further information.

Data

Policy information about availability of data

All manuscripts must include a data availability statement. This statement should provide the following information, where applicable:

- Accession codes, unique identifiers, or web links for publicly available datasets
- A list of figures that have associated raw data
- A description of any restrictions on data availability

A data availability section has been added to the manuscript. Additional data that support the findings of this study are available from the corresponding author

April 2020

upon reasonable request.

Field-specific reporting

Please select the one below that is the best fit for your research. If you are not sure, read the appropriate sections before making your selection.

☒ Life sciences ☐ Behavioural & social sciences ☐ Ecological, evolutionary & environmental sciences

For a reference copy of the document with all sections, see nature.com/documents/nr-reporting-summary-flat.pdf

Life sciences study design

All studies must disclose on these points even when the disclosure is negative.

Sample size	Sample sizes used in the study were designed based on the sample size required to produce robust statistics from the relevant statistical methods used in each experiment. Sample sizes were chosen for the differing experimental approaches based on the technical difficulty and throughput of the individual assays. For the SERBP1 M-bodies quantification, N=15 or N=20 mitotic cells were enough to detect a significant difference. For the quantification of binucleated cells and PICH- or DAPI-positive bridges we quantified as previously (see Kelly J. et al, Nat Comms, 2020).
Data exclusions	No data have been excluded from the analysis.
Replication	For the in cell data presented, a minimum of 3 independent experimental repeats were performed using to ensure reproducibility of the results. The exception to this are the in vitro experiments (2 independent experiments), the mass spectrometry and peptide array assays, as they were used as a screening tool, the results of which were validated in subsequent experiments.
Randomization	Randomization was not applicable to the study as all experiments were performed using a small number of samples and treatments. The assays used in this study (drug treatment or protein downregulation) are not subject to the systematic variation which demands randomization. We considered blinding as more appropriate for the type of assays used in this study.
Blinding	Blinding was used for quantification of binucleated cells, SERBP1 M-bodies and PICH- or DAPI-positive bridges in which scoring was performed manually. Individual images from the data sets were assigned random numbers by a colleague who was blinded during the data collection and re-sorted into the appropriate experimental groups post analysis. For experiments where data scoring/analysis was performed using automated means and pre-defined software (as using Image J for immunoblotting quantification) blinding was not deemed necessary.

Reporting for specific materials, systems and methods

We require information from authors about some types of materials, experimental systems and methods used in many studies. Here, indicate whether each material, system or method listed is relevant to your study. If you are not sure if a list item applies to your research, read the appropriate section before selecting a response.

Materials & experimental systems

n/a	Involved in the study
☐	☒ Antibodies
☐	☒ Eukaryotic cell lines
☒	☐ Palaeontology and archaeology
☒	☐ Animals and other organisms
☒	☐ Human research participants
☒	☐ Clinical data
☒	☐ Dual use research of concern

Methods

n/a	Involved in the study
☒	☐ ChIP-seq
☒	☐ Flow cytometry
☒	☐ MRI-based neuroimaging

Antibodies

Antibodies used

rabbit anti PKCe - sc214, Santa Cruz
rabbit anti-PKCe phosphoSer729 - polyclonal, 44-977G, Invitrogen
rabbit anti-PKCe phosphoThr566 - polyclonal, made in-house
rabbit anti-PKCe phosphoThr710 - polyclonal, made in-house
rabbit anti-PKC pSer substrate - 2261, Cell Signalling Technology
mouse anti-alpha tubulin, made in-house
rabbit anti-PICH - H00054821-D01, Abnova
mouse anti-Lap2B, 611000, BD
mouse anti-GAPDH (clone 6C5) - MAB374, Millipore
mouse anti-Myc - 2276, Cell Signalling Technology
mouse anti-FLAG M2- monoclonal, B3111 Sigma
rabbit anti-SERBP1 - polyclonal, 55993 Abcam

mouse anti-PABP F-2 - monoclonal, sc-166027 Santa Cruz
mouse Anti-Fragile X Mental Retardation Protein Antibody, clone 1C3 - MAB2160 Millipore
rabbit anti-LARP4B - polyclonal, NBP1-80890 Novus Biologicals
mouse Anti-Puromycin Antibody, clone 17H1 - monoclonal, MABE341 Millipore
rabbit EGF Receptor (D38B1) - monoclonal, #4267 Cell Signaling
anti-mouse HRP conjugated secondary antibody - NA931, GE Lifesciences
anti-rabbit HRP conjugated secondary antibody - NA934, GE Lifesciences
Clone 35/GM130 mouse anti-G3BP - monoclonal, 610823 BD Biosciences
S6 Ribosomal Protein (5G10) - monoclonal, 2217 Cell Signaling
mouse anti-phospho Ser/Thr-pro MPM2 - monoclonal, 05-368 Millipore
mouse anti-Vimentin (V9) - monoclonal, sc-6260 Santa Cruz
Goat anti-mouse AlexaFluor 488 Conjugated secondary antibody - A11001, Life technologies
Goat anti-rabbit AlexaFluor 488 Conjugated secondary antibody - A11008, Life technologies
Goat anti-mouse AlexaFluor 555 Conjugated secondary antibody - A21422, Life technologies
Goat anti-rabbit AlexaFluor 555 Conjugated secondary antibody - A21428, Life technologies

Validation

rabbit anti PKCe - sc214, Santa Cruz (https://www.scbt.com/p/pkc-epsilon-antibody-c-15)

rabbit anti-PKCe phosphoThr566 - polyclonal, made in-house (see Cameron A et al., Nature Structural and Molecular Biology, 2009)

rabbit anti-PKCe phosphoThr710 - polyclonal, made in-house (see Cameron A et al., Nature Structural and Molecular Biology, 2009)

rabbit anti-PKC pSer substrate - 2261, Cell Signalling Technology (see Cameron A et al., Nature Structural and Molecular Biology, 2009)

rabbit anti-PICH - H00054821-D01, Abnova (http://www.abnova.com/products/products_detail.asp?catalog_id=H00054821-D01)

mouse anti-Lap2B, 611000, BD (https://www.bdbiosciences.com/us/reagents/research/antibodies-buffers/cell-biology-reagents/cell-biology-antibodies/purified-mouse-anti-lap2-27lap2/p/611000)

mouse anti-alpha tubulin, made in-house

mouse anti-GAPDH - MAB374, Millipore (https://www.merckmillipore.com/GB/en/product/Anti-Glyceraldehyde-3-Phosphate-Dehydrogenase-Antibody-clone-6C5,MM_NF-MAB374)

mouse anti-Vimentin (V9) - monoclonal, sc-6260 Santa Cruz (https://www.scbt.com/p/vimentin-antibody-v9)

mouse anti-phospho Ser/Thr-pro MPM2 - monoclonal, 05-368 Millipore (see Deiss et al., Nucleic Acid Research, 2018)

S6 Ribosomal Protein (5G10) - monoclonal, 2217 Cell Signaling (https://www.cellsignal.com/products/primary-antibodies/s6-ribosomal-protein-5g10-rabbit-mab/2217)

Clone 35/GM130 mouse anti-G3BP - monoclonal, 610823 BD Biosciences (https://www.bdbiosciences.com/eu/reagents/research/antibodies-buffers/cell-biology-reagents/cell-biology-antibodies/purified-mouse-anti-gm130-35gm130/p/610823)

rabbit EGF Receptor (D38B1) - monoclonal, #4267 Cell Signaling (https://www.cellsignal.com/products/primary-antibodies/egf-receptor-d38b1-xp-rabbit-mab/4267)

mouse Anti-Puromycin Antibody, clone 17H1 - monoclonal, MABE341 Millipore (https://www.merckmillipore.com/GB/en/product/Anti-Puromycin-Antibody-clone-17H1,MM_NF-MABE341?ReferrerURL=https%3A%2F%2Fwww.google.com%2F&bd=1)

rabbit anti-LARP4B - polyclonal, NBP1-80890 Novus Biologicals (https://www.novusbio.com/products/larp4-antibody_nbp1-80890)

mouse Anti-Fragile X Mental Retardation Protein Antibody, clone 1C3 - MAB2160 Millipore (https://www.merckmillipore.com/GB/en/product/Anti-Fragile-X-Mental-Retardation-Protein-Antibody-clone-1C3,MM_NF-MAB2160?ReferrerURL=https%3A%2F%2Fwww.google.com%2F&bd=1)

mouse anti-PABP F-2 - monoclonal, sc-166027 Santa Cruz (https://www.scbt.com/p/pabp-antibody-f-2)

rabbit anti-SERBP1 - polyclonal, 55993 Abcam (https://www.abcam.com/serbp1-antibody-ab55993.html)

mouse anti-FLAG M2- monoclonal, B3111 Sigma (see Elbediwy et al., Journal of Cell Science, 2019)

mouse anti-Myc - 2276, Cell Signalling Technology (https://www.cellsignal.com/products/primary-antibodies/myc-tag-9b11-mouse-mab/2276?_=1594366677542&Ntt=myc&tahead=true)

Eukaryotic cell lines

Policy information about cell lines

Cell line source(s)	DLD1, HeLa and HEK293T cell lines were obtained by the Cell Services Science Technology Platform at the Francis Crick Institute.<br>DLD1-FRT-Trex were kindly provided by Prof. Stephen Taylor.
Authentication	All cell lines were authenticated by the Francis Crick Institute by STR profiling
Mycoplasma contamination	Cell lines were routinely tested for mycoplasma. No contamination found.
Commonly misidentified lines (See ICLAC register)	No commonly misidentified cell lines were used in this study.
