# Supplementary material for: A genetically-encoded crosslinker screen identifies SERBP1 as a PKCε substrate influencing translation and cell division
Source: Nat Commun. 2021 Nov 26;12:6934. doi: 10.1038/s41467-021-27189-5 (PMC8626422; doi:10.1038/s41467-021-27189-5)
Supplement: Supplementary file 5 — Source Data [file 41467_2021_27189_MOESM5_ESM.pdf]

Figure 1e-left

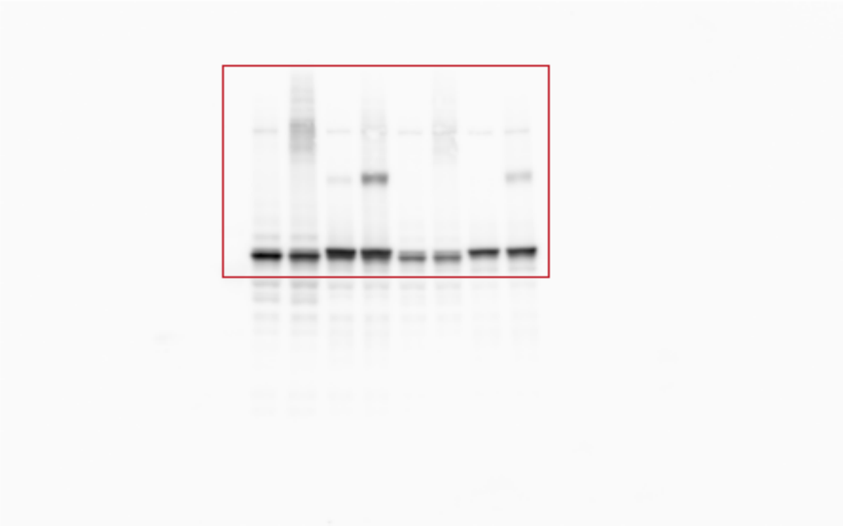

Figure 1e-right

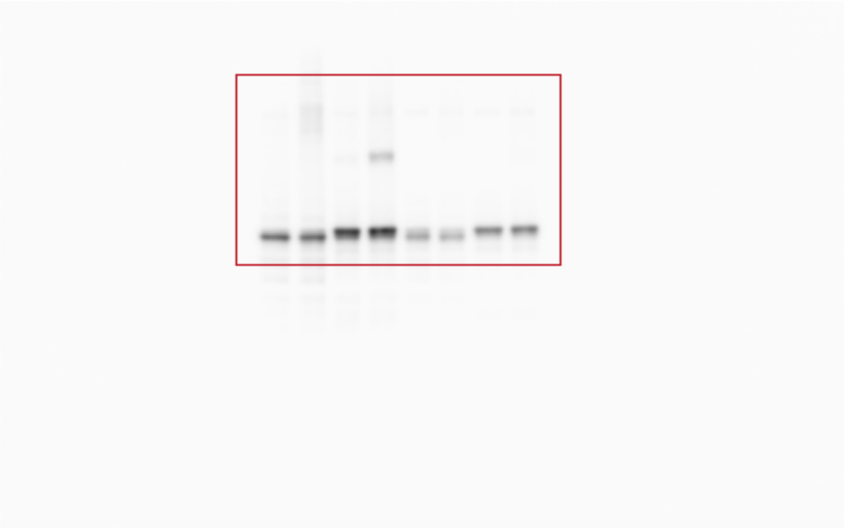

Figure 2a

SERBP1

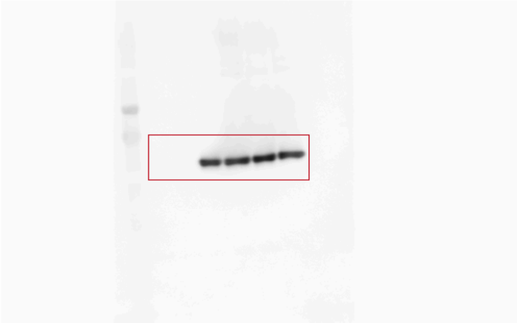

Figure 2b upper panel

SERBP1

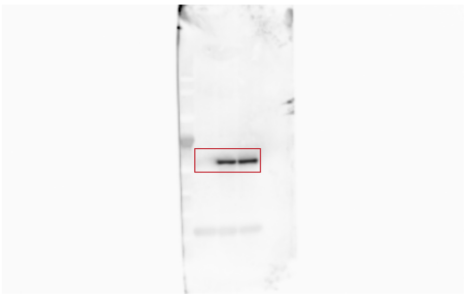

ProQ

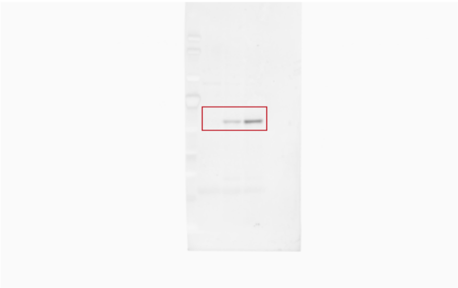

Figure 2d

ProQ

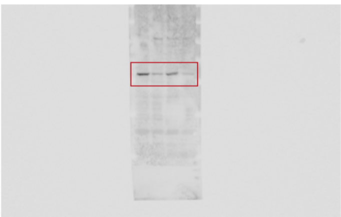

FLAG

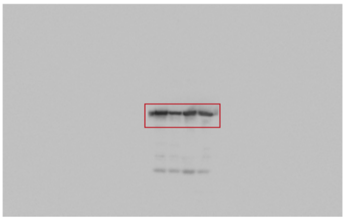

PKCε

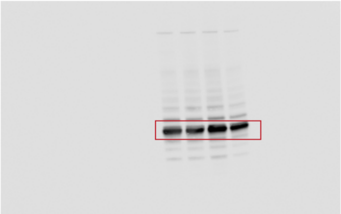

Figure 2e

FLAG

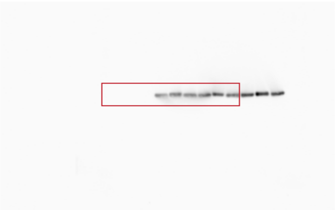

ProQ

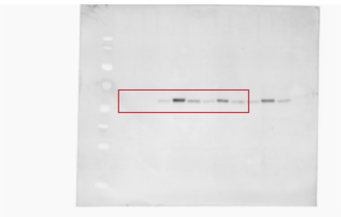

PKCε

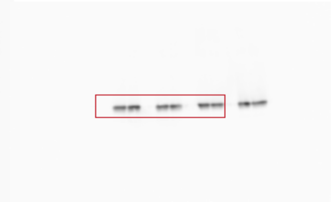

Figure 2e - PROQ-diamond staining normalized to anti-FLAG

	SERBP1-WT	RBP1-WT + PK	P1-WT + PKCε + Bl	SERBP1 S74A	SERBP1 S74A + PKCε	RBP1 S74A + PKCε + Blu577
EXP1	0.12854547	0.85853024	0.23325988	0.13844782	0.4555324	0.17462433
EXP2	0.16160957	0.82044224	0.27629134	0.08503147	0.37257895	0.15218103

Figure 2d - PROQ-diamond staining normalized to anti-FLAG

	SERBP1-WT	SERBP1 S74A	SERBP1 S386A	SERBP1 S74/386A
EXP1	0.59891731	0.16175403	0.47104484	0.1301142
EXP2	0.47708552	0.30090409	0.59228124	0.31377558
EXP3	0.59760072	0.421441	0.59363402	0.50758071
EXP4	0.93859668	0.45917858	0.75792603	0.40025288

Figure 2b

	SERBP1	BP1+PKCε E612Abk
EXP1	1.19644088	2.30897971
EXP2	0.80355912	1.96679225

Figure 3g

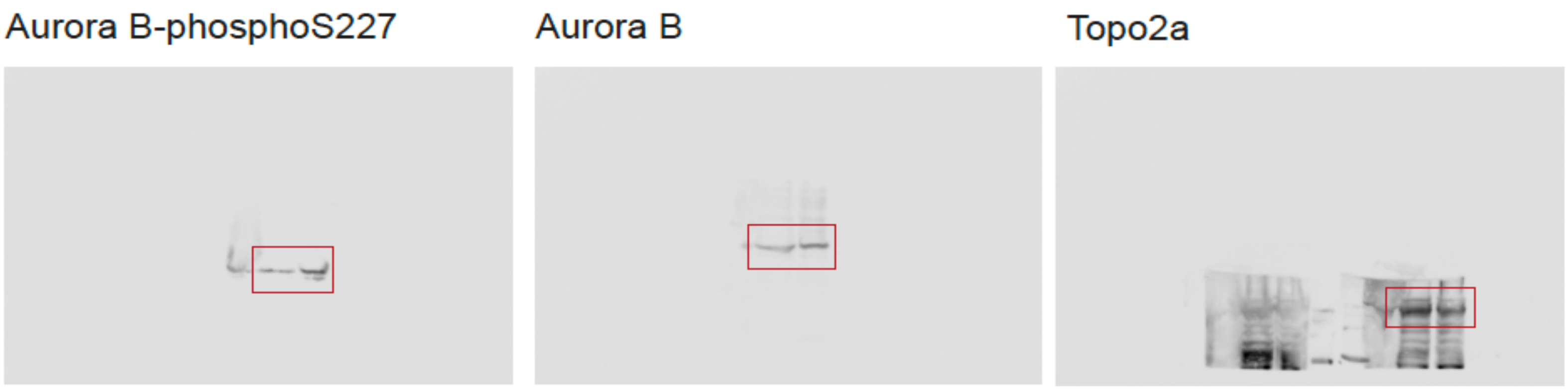

Figure 3d-Number of foci per cell

	siControl	EV	SERBP1-WT	SERBP1-S74A
EXP 1	1	0.154381	0.40867161	0.12947198
EXP 2	1	0.11	0.406675	0.081675
EXP 3	1	0.028903	0.69719388	0.07091837

Figure 3e-Number of foci per cell

	DLD1 PAR		DLD1 eD383/451N	
	siControl	siPKCε		
EXP 1	1	0.153061	1	0.4635462
EXP 2	1	0.218367	1	0.04380044
EXP 3	1	0	1	0.2181031

Figure 3c-Number of foci per cell

	DMSO	Blu577
EXP 1	1	0.104662
EXP 2	1	0.286319
EXP 3	1	0.329167

DLD1 PAR

	siControl	siPKCε
EXP 1	1	0.2183
EXP 2	1	0
EXP 3	1	0.153

DLD1-εWT

	siControl	siPKCε
EXP 1	1	1.067905
EXP 2	1	0.310714
EXP 3	1	0.741619

	DMSO	Blu577	ZM447439
EXP 1	1	0.295964	1.034316361
EXP 2	1	0.405841	0.856514413
EXP 3	1	0.04932	0.53846154

Figure 4d

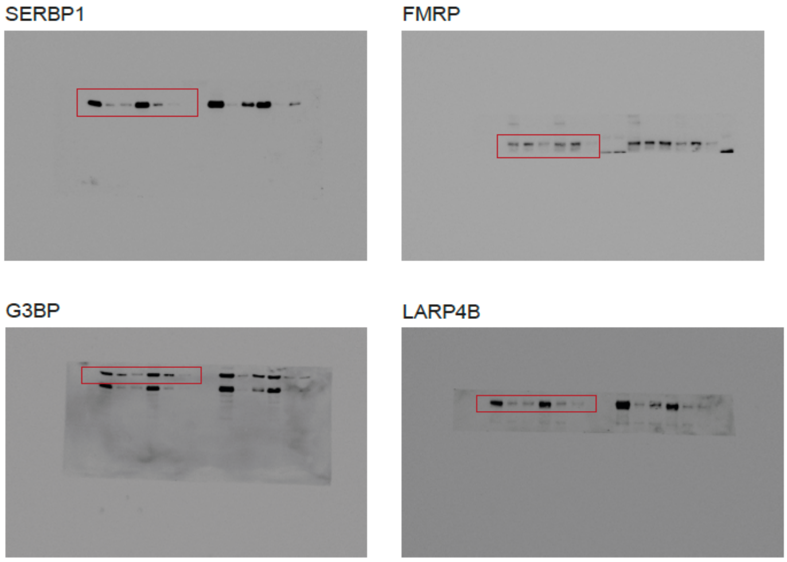

Figure 4b-upper Intensity (*10^4)

Distance (um)	DAPI	SERBP1	PABP
0	1389	3175	735
0.132	1418	3194	975
0.264	1369	2954	1596
0.396	1402	3279	2177
0.528	1385	2947	1476
0.66	2043	2971	2101
0.791	1153	3124	1412
0.923	1109	3110	892
1.055	1257	3246	1294
1.187	1096	3004	1051
1.319	1392	2950	1706
1.451	1387	3087	1268
1.583	1312	3182	1286
1.715	2150	3453	1556
1.847	1762	3470	1215
1.979	1252	3583	2453
2.111	1318	4506	3853
2.243	1915	4176	5189
2.374	1336	3730	2986
2.506	2102	3191	2299
2.638	1239	3027	2680
2.77	1438	2912	4644
2.902	1164	3494	8507
3.034	2237	4032	6872
3.166	1526	3681	2943
3.298	1749	4296	2412
3.43	1451	3315	2357
3.562	1385	4509	2940
3.694	1804	3236	3833
3.826	1392	3901	3506
3.957	1486	4522	1954
4.089	2073	4314	3131
4.221	2098	3360	1643
4.353	1200	3099	2602
4.485	1266	3611	4653
4.617	1714	4843	3921
4.749	1637	4129	3009
4.881	1310	5886	4206
5.013	1714	5417	5300
5.145	1926	4076	5603
5.277	1484	5796	7917
5.409	1458	8153	5103
5.54	1894	10965	10337
5.672	1566	9436	8057
5.804	2298	5611	5306
5.936	2027	9356	5755
6.068	1570	5593	4279
6.2	1736	5018	7400
6.332	1816	3890	2614
6.464	1705	4849	5588
6.596	1645	6274	4173
6.728	2393	5711	7816
6.86	1917	4333	3989
6.991	1844	5026	3127
7.123	1617	5034	4855
7.255	1777	5963	5260
7.387	1864	4285	4380
7.519	1447	5559	9390
7.651	2082	8397	9840
7.783	1919	8359	10496
7.915	2294	6319	6372
8.047	2360	5144	3994
8.179	2398	4364	4211
8.311	1687	4645	5361
8.443	2605	5252	5372
8.574	1923	6149	8742
8.706	2223	4661	8350
8.838	1457	4476	3805
8.97	1901	4893	5490
9.102	2020	4266	3648
9.234	1632	4089	3526
9.366	1674	4949	5807

Figure 4b-lower panel

Distance (um)	DAPI	SERBP1	PABP
0	3594	4677	9752
0.066	5032	4160	7755
0.132	3605	4575	7396
0.198	3900	4831	10775
0.264	3544	5248	10544
0.33	4081	6306	11822
0.396	4025	5979	12402
0.462	3647	5589	9756
0.528	4306	5669	10180
0.594	4228	6193	14934
0.66	4392	6267	10358
0.726	4089	5923	14559
0.792	3942	5888	12574
0.858	3307	5626	13678
0.924	3307	5626	13678
0.99	4415	6933	11236
1.056	3886	7459	12027
1.122	4267	7566	10767
1.188	4137	6797	12885
1.254	3625	6864	8878
1.32	4089	6578	10514
1.386	3803	5747	12357
1.452	5035	6048	10161
1.518	4651	6801	12914
1.584	5280	5737	11524
1.65	3630	6126	12261
1.716	3630	6126	12261
1.782	4144	5959	13947
1.848	3652	6245	11727
1.914	4267	6198	12624
1.98	4162	6044	11589
2.046	3883	6457	11157
2.112	5823	5629	10890
2.178	4089	6295	9955
2.244	4735	5898	12257
2.31	4735	5898	12257
2.376	3944	6205	11927
2.442	5534	6471	10197
2.508	4565	6566	9429
2.574	4854	6160	12726
2.64	4624	6045	9452
2.706	4172	5614	9130
2.772	5339	6041	8742
2.838	5339	6041	8742
2.904	4679	6339	10418
2.97	4899	5406	10036
3.036	3889	5565	9959
3.102	4145	6134	9454
3.168	4420	5384	13960
3.234	4395	5998	9124
3.3	4654	6366	10099
3.366	5369	5396	9963
3.432	4448	5707	10541
3.498	4359	7114	10560
3.564	4312	6018	11825
3.63	4312	6018	11825
3.696	5428	5654	12324
3.762	4916	8999	10637
3.828	6036	8256	10309
3.894	6616	12686	9926
3.96	10534	16874	8669
4.026	11608	11079	12545
4.092	14938	8486	12716
4.158	13302	10752	9667
4.224	15423	7718	11516
4.29	14963	5382	8972
4.356	19327	5827	9303
4.422	19327	5827	9303
4.488	19235	5204	9794
4.554	20751	4987	9136
4.621	18701	5018	8888
4.687	23283	4775	9230

Figure 4c

Distance (um	DAPI	SERBP1	FMRP
0	1427	13899	17029
0.092	1267	17009	25911
0.184	1249	15031	23385
0.276	1714	11923	17916
0.368	1867	9473	13913
0.46	2016	7710	11676
0.553	1393	7655	10747
0.645	1929	9052	9870
0.737	1343	10779	11561
0.829	1223	11361	11558
0.921	1144	9112	14196
1.013	1539	10866	13601
1.105	1849	11056	14882
1.197	1601	12077	17127
1.289	1289	11159	16064
1.381	1732	12286	18405
1.473	1239	13516	22503
1.565	1579	17050	26484
1.658	1558	26295	32936
1.75	1642	35638	47680
1.842	1393	32262	40591
1.934	1258	32972	35206
2.026	1275	26194	32556
2.118	1787	18060	21926
2.21	1183	16037	13778
2.302	1402	15377	12827
2.394	1201	13257	13497
2.486	1335	12278	13157
2.578	1619	11646	11893
2.67	1442	10680	12169
2.763	1444	12389	15494
2.855	1352	12943	14808
2.947	1482	13273	16104
3.039	1575	12423	15940
3.131	1312	10912	16451
3.223	1179	10487	13901
3.315	1774	11605	14209
3.407	1734	11879	12055
3.499	1300	11757	12469
3.591	2055	11068	11529
3.683	1264	11979	13192
3.775	2016	11963	13738
3.868	1815	11516	13267
3.96	2267	11336	13144
4.052	1989	10554	12952
4.144	1501	12149	15415
4.236	1446	12033	17720
4.328	1909	12179	17486
4.42	1456	13070	15582
4.512	1339	11960	15217
4.604	1618	10581	16014
4.696	1416	10298	13629
4.788	1828	11014	13097
4.88	1284	9244	12854
4.973	1359	8989	12273
5.065	2120	7157	9859
5.157	1406	7669	8969
5.249	1500	7736	8197
5.341	1212	7641	10580
5.433	1436	8124	10644
5.525	1939	9975	11174
5.617	1431	11275	11654
5.709	1135	10943	8905
5.801	1743	11667	11517
5.893	1060	12061	12700
5.985	1034	13110	12298
6.078	1981	14869	18424
6.17	1243	17038	20254
6.262	2124	19677	17170
6.354	1388	16594	15740
6.446	1439	13384	16886
6.538	1947	9799	14820

9.498	1458	6822	7730
9.63	2294	8279	8559
9.762	1798	11084	10368
9.894	2217	14955	10406
10.026	2054	12699	8231
10.157	1861	7586	8496
10.289	2490	5887	9443
10.421	2208	6798	11249
10.553	1923	5490	5320
10.685	2327	6071	6646
10.817	2621	6441	7937
10.949	1881	4783	5340
11.081	1782	5002	4702
11.213	2003	6013	4211
11.345	2396	5231	4562
11.477	2047	6093	6689
11.609	2263	5941	5699
11.74	2618	8823	10500
11.872	2985	11421	15822
12.004	2537	14593	16949
12.136	2420	18067	20722
12.268	2393	20619	16972
12.4	2698	17652	17768
12.532	2747	15200	13739
12.664	1983	10696	13582
12.796	3199	8475	6854
12.928	2954	6743	6022
13.06	2552	5963	8421
13.192	2232	4821	6324
13.323	2773	4864	5720
13.455	3060	5458	6374
13.587	2565	5107	6022
13.719	1707	5129	5815
13.851	1670	4421	6921
13.983	2808	4196	6586
14.115	2608	5072	10479
14.247	2619	6102	10444
14.379	2685	6542	7146
14.511	2016	7061	5525
14.643	2146	4894	9006
14.774	3155	6118	8893
14.906	2433	6668	9491
15.038	3287	7068	7916
15.17	2703	5580	10894
15.302	2120	5944	7910
15.434	2758	5500	7297
15.566	3866	5864	5533
15.698	2839	5442	6399
15.83	2300	5620	8289
15.962	3576	6706	8853
16.094	2568	7213	6467
16.226	2263	6379	7323
16.357	2449	6189	8587
16.489	2954	6734	7213
16.621	3437	6230	9650
16.753	2493	6584	6954
16.885	2685	7726	11950
17.017	2594	6658	8241
17.149	2036	7301	6055
17.281	2457	7666	4628
17.413	3016	11616	9837
17.545	2938	13267	15848
17.677	2583	8427	13119
17.809	3857	6118	7884
17.94	4078	6643	6596
18.072	6084	5502	6544
18.204	5477	7313	6406
18.336	9925	5492	5505
18.468	8860	5592	5628
18.6	10157	5052	4898
18.732	10067	4455	5829
18.864	11077	4434	4400
18.996	11186	4887	4256
19.128	11563	4360	8238
19.26	9767	4561	6779
19.392	11773	5119	6404
19.523	8996	4938	8253
19.655	9276	5498	7628
19.787	7474	4941	5204
19.919	15041	4533	4348
20.051	9963	3769	6079
20.183	9029	4580	4270
20.315	7941	4100	4989
20.447	10930	4046	9192
20.579	11046	4590	7865
20.711	10504	4897	5626
20.843	8436	4004	5085
20.974	9840	3842	6280
21.106	12940	3535	7551
21.238	11914	3768	8148
21.37	11135	3522	5529
21.502	11373	3993	5146
21.634	10570	4192	6144
21.766	10336	5266	7001
21.898	10623	5367	4982
22.03	10468	6365	6929
22.162	11139	7664	7012
22.294	10970	4768	7448
22.426	8473	4765	11406
22.557	11989	5377	10702
22.689	11989	5377	10702
22.821	11314	4743	5106
22.953	14077	4060	4886
23.085	14141	4425	4611
23.217	15547	4030	5718
23.349	13273	3594	7029
23.481	12803	3523	4112
23.613	10890	4551	5065
23.745	11089	4219	5343
23.877	11025	4946	6743
24.009	8529	5159	6925
24.14	12271	3871	8660
24.272	10581	4019	7690

4.753	21144	5385	9216
4.819	19009	5103	10512
4.885	24528	5020	10038
4.951	23965	5599	7440
5.017	20598	5280	9477
5.083	23818	5649	8794
5.149	18920	5299	11142
5.215	20595	5557	8331
5.281	26848	5892	8838
5.347	19827	5181	7342
5.413	24299	4552	7125
5.479	25922	4711	6413
5.545	25922	4711	6413
5.611	23935	4927	7744
5.677	24151	4663	7382
5.743	20273	4307	6613
5.809	22385	4411	7302
5.875	22357	4325	5979
5.941	20198	4338	5860
6.007	20835	3859	7419
6.073	19674	3990	6002
6.139	20595	3884	6789
6.205	22006	3936	6129
6.271	21667	3866	7194
6.337	21667	3866	7194
6.403	21522	3984	8824
6.469	19152	3888	8552
6.535	21019	3650	8047
6.601	20637	3627	7133
6.667	22001	4465	6159
6.733	22028	3573	7634
6.799	23197	3750	5222
6.865	22585	3544	4747
6.931	22162	3775	4082
6.997	23637	3770	4017
7.063	22357	4315	3556
7.129	22357	4315	3556
7.195	20303	4079	4349
7.261	21483	4145	3220
7.327	20367	3760	4587
7.393	23428	3947	4497
7.459	22252	4453	4241
7.525	20167	4445	5843
7.591	21238	4855	5419
7.657	19009	5453	6774
7.723	23222	5572	7117
7.789	21046	4653	7000
7.855	20242	4435	6902
7.921	23812	4710	9529
7.987	21330	4630	6991
8.053	20109	4312	6802
8.119	19366	5843	7505
8.185	18353	7884	7255
8.251	18353	7884	7255
8.317	18392	12659	7463
8.383	20059	20733	6897
8.449	21572	19584	7133
8.515	19477	24473	8535
8.581	17880	20800	9076
8.647	19833	15462	7363
8.713	20782	10469	9667
8.779	18804	7985	8843
8.845	18812	6493	8114
8.911	18228	5731	8425
8.977	15400	5448	7209
9.043	15400	5448	7209
9.109	13238	5246	10683
9.175	11302	5755	11109
9.241	9371	5585	8599
9.307	12631	5450	8882
9.373	9885	5333	10800
9.439	9443	5143	8296
9.505	8895	5026	7694
9.571	8895	5026	7694
9.637	8344	5433	10996
9.703	7865	5441	10808
9.769	6811	5426	8006
9.835	8274	5423	8381
9.901	8589	5729	8970
9.967	9838	5721	10253
10.033	5694	5631	10057
10.099	6310	5622	12618
10.165	6310	5622	12618
10.231	5628	5862	13023
10.297	5814	6361	15903
10.363	4050	6987	10959
10.429	4971	6030	12146
10.495	4609	7211	12292
10.561	4401	6696	10829
10.627	4429	6296	14308
10.693	4743	7048	10647
10.759	4194	7120	14469
10.825	4312	6884	9326
10.891	4721	6582	10149
10.957	4721	6582	10149
11.023	4164	7029	9782
11.089	4885	6728	11384
11.155	3906	6506	10610
11.221	4306	5990	11330
11.287	4159	6214	10919
11.353	4270	6461	13576
11.419	4551	5715	14448
11.485	4935	6162	11351
11.551	4840	5871	13021
11.617	4362	6138	10875
11.683	4125	6165	13210
11.749	4417	6161	12261
11.815	4016	5837	12039
11.881	4303	5816	11867
11.947	4279	6090	9494
12.013	4910	6308	14590
12.079	4289	5810	13060
12.145	4248	6550	14237

6.63	2073	8719	14685
6.722	1274	7744	14097
6.814	1290	6395	11846
6.906	1788	6234	12519
6.998	1333	6281	9982
7.09	1501	6332	6961
7.183	1520	7092	7969
7.275	1500	7532	6670
7.367	1524	7339	6577
7.459	1693	7806	6203
7.551	1204	8441	8402
7.643	1447	8487	6976
7.735	1487	7307	7022
7.827	1448	5829	6306
7.919	1228	5499	6648
8.011	1351	6140	7270
8.103	1513	8420	9234
8.195	1679	12035	15128
8.288	1137	15699	19481
8.38	1158	19305	24749
8.472	1747	18670	26277
8.564	1475	16647	22041
8.656	1593	14563	19321
8.748	1503	11454	17479
8.84	1650	9009	13737
8.932	1617	9092	13857
9.024	1128	9060	10333
9.116	1389	8389	11087
9.208	1729	8674	7827
9.301	1180	8793	7211
9.393	1176	8443	7392
9.485	1784	8935	8967
9.577	1377	8941	9083
9.669	1783	8691	9740
9.761	1371	8501	8640
9.853	1428	7011	8060
9.945	1829	7203	9077
10.037	1055	9097	8539
10.129	1553	10574	7264
10.221	1421	8930	8076
10.313	1646	8280	9184
10.406	1569	9458	7057
10.498	1175	8222	6136
10.59	1655	6722	6827
10.682	1303	7499	6721
10.774	1176	7967	7090
10.866	1152	6429	5729
10.958	1242	5854	5901
11.05	1137	6245	5435

24.404	10427	4374	4472
24.536	9864	5466	6489
24.668	11424	4036	4873
24.8	9146	3643	5311
24.932	10332	4686	3820
25.064	8871	4159	6373
25.196	12507	4788	6548
25.328	10687	3798	5191
25.46	11018	4741	4409
25.592	12177	4215	4600
25.723	10338	3409	3888
25.855	8127	5623	7314
25.987	7613	5171	6723
26.119	7906	6344	5540
26.251	5112	6425	9380
26.383	4620	6629	8165
26.515	7017	5350	6674
26.647	5823	4716	5705
26.779	8063	4412	5229
26.911	8105	3852	9429
27.043	8723	3881	10004
27.174	6145	3648	9183
27.306	6172	3541	5978
27.438	6917	3382	8718
27.57	7306	3888	4596
27.702	7017	4399	4910
27.834	9974	3603	5197
27.966	7138	3321	5219
28.098	7489	3703	5156
28.23	8272	3649	4982
28.362	8513	3536	5300
28.494	7261	3354	5722
28.626	7964	3425	5149
28.757	10031	2922	3350
28.889	10808	3282	4689
29.021	10164	3246	4105
29.153	11888	3654	6303
29.285	9705	3595	6594
29.417	9656	3700	3817
29.549	10782	3471	4379
29.681	9014	4029	5934
29.813	8262	3402	4737
29.945	8553	3459	7293
30.077	8125	3725	7271
30.209	8361	3783	7218
30.34	9756	3441	5750
30.472	8206	3398	3259
30.604	9005	3571	3707
30.736	7531	3405	3524
30.868	7849	3645	6988
31	9520	4038	5207
31.132	13536	3463	5247
31.264	11781	3732	4815
31.396	10881	3568	4334
31.528	12925	3072	5201
31.66	12686	3191	3764
31.792	11088	3976	3580
31.923	10100	3528	3335
32.055	10656	3695	4401
32.187	11214	3112	4129
32.319	10894	3464	4058
32.451	12347	3194	5394
32.583	10166	3528	4565
32.715	11742	3233	6343
32.847	7641	3151	6320
32.979	9656	3187	5752
33.111	8886	3388	5949
33.243	6986	3318	4457
33.374	7043	2958	5196
33.506	9394	3244	6642
33.638	10074	3099	5967
33.77	8842	3480	5280
33.902	5993	3145	5106
34.034	6534	3772	5498
34.166	6929	3387	5164
34.298	6944	4579	4259
34.43	7251	6901	4801
34.562	7202	5783	4631
34.694	6635	4967	4004
34.826	4512	3514	3113
34.957	6022	3195	4166
35.089	6432	3272	5388
35.221	5154	3415	5330
35.353	5337	3274	4893
35.485	5373	3472	4964
35.617	6702	3648	5104
35.749	8008	4652	3550
35.881	6898	5020	3934
36.013	12887	5304	3640
36.145	10268	5119	8350
36.277	10073	5590	8387
36.409	8504	4534	5701
36.54	9464	4080	4452
36.672	8950	4418	6960
36.804	8118	3920	5929
36.936	10780	3706	4762
37.068	9572	3815	6121
37.2	9091	3700	6245
37.332	7906	3934	4505
37.464	9767	3995	5901
37.596	10312	4236	7690
37.728	10118	4304	5934
37.86	8915	3861	3700
37.992	7498	3650	5834
38.123	9226	3860	4327
38.255	7127	3759	5328
38.387	9101	3688	4134
38.519	8222	4107	4437
38.651	10742	3783	5401
38.783	8372	3864	4107
38.915	8328	4283	4422
39.047	7326	4303	5811
39.179	7864	4598	9595

12.211	3872	5777	12200
12.277	3872	5777	12200
12.343	4220	5902	10531
12.409	4312	6252	12350
12.475	4810	6594	11695
12.541	3752	6381	12432
12.607	3911	6829	12423
12.673	4768	6545	13759
12.739	4298	6424	10894
12.805	5099	7253	11194
12.871	5099	7253	11194
12.937	3761	9834	10017
13.003	3919	15571	11514
13.069	5544	16235	12267
13.135	3454	16020	12793
13.201	3549	10776	10360
13.267	4245	7133	11852
13.333	3388	6606	10677
13.399	3152	5898	10455
13.465	4473	6011	8945
13.531	2965	4773	9782
13.597	2731	4815	7807

39.311	5514	6168	5899
39.443	4016	4740	6785
39.575	2307	5540	8615
39.706	2184	5346	12599
39.838	3080	4554	12857
39.97	2678	4418	9233
40.102	1499	4137	3729
40.234	2356	5222	6387
40.366	2214	4690	4850
40.498	2212	4486	9585
40.63	2857	4074	6061
40.762	2027	5395	10166
40.894	2058	6757	8543
41.026	2945	5939	6111
41.157	1895	5608	8635
41.289	2561	5252	7869
41.421	2848	4372	7412
41.553	2718	4522	3840
41.685	2404	4392	3858
41.817	2261	4387	4942
41.949	2281	3732	5042
42.081	2257	4584	3850
42.213	1954	4590	4817
42.345	1621	5575	4231
42.477	1890	4509	8549
42.609	2009	4782	4249
42.74	2149	4541	5962
42.872	2619	4912	12410
43.004	1742	5469	7881
43.136	2283	8502	5535
43.268	2102	9628	6674
43.4	2338	6488	9224
43.532	2808	6936	8401
43.664	1705	5431	4566
43.796	2738	4798	4156
43.928	2170	4729	5578
44.06	2745	3815	9458
44.192	2630	3900	8180
44.323	1901	4397	8291
44.455	1517	5195	3926
44.587	3369	3807	3128
44.719	1850	3565	3415
44.851	1643	4140	5002
44.983	1663	4938	8936
45.115	2049	5202	7713
45.247	1742	4642	7510
45.379	1431	3778	4583
45.511	1811	3638	3333
45.643	1937	3451	3334

Figure 6

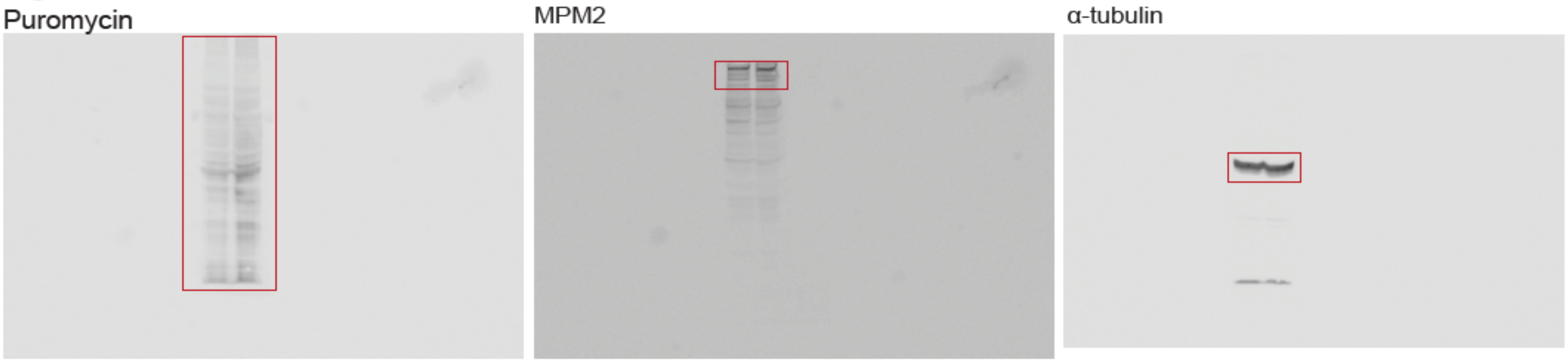

Figure 4d

	DMSO	Puromycin	Blu577	Puromycin + Blu577
EXP 1	1	3.683502	0.05387205	1.62626263
EXP 2	1	2.2	0.0557377	1.40655738
EXP 3	1	5.083682	0.24476987	2.48953975

Figure 4b

	DMSO	Puromycin	Cycloheximide
EXP 1	1	4.163934	0.49180328
EXP 2	1	7.836207	0.13793103
EXP 3	1	8.085366	0.36585366

Figure 6f - anti-Puromycin normalized to anti-αTubulin

	DMSO	Blu577
EXP 1	1.487845	3.272974
EXP 2	1.496545	3.013563
EXP 3	1.620959	2.692285

Figure 7b - % binucleated cells

	siControl	siSerbp1
EXP 1	8.77193	26.04651
EXP 2	7.761194	23.56495
EXP 3	6.970509	17.29107

Figure 7a

PICH-positive strands (% anaphase cells)

	siControl	siSerbp1
EXP 1	10	26.66667
EXP 2	18.18	37.5
EXP 3	20	50

DAPI-positive bridges (% anaphase cells)

	siControl	siSerbp1
EXP 1	5.882353	28.57143
EXP 2	8.333333	18.18182
EXP 3	6.666667	16.66667

Figure 7c - % binucleated cells

	siControl	EV	SERBP1-WT	SERBP1-S74A
EXP 1	2.005731	7.467532	2.30263158	3.21428571
EXP 2	1.369863	4.255319	2.31660232	5.24344569
EXP 3	1.683928	6.302958	1.904857394	5.78493762

Supplementary Figure 1d

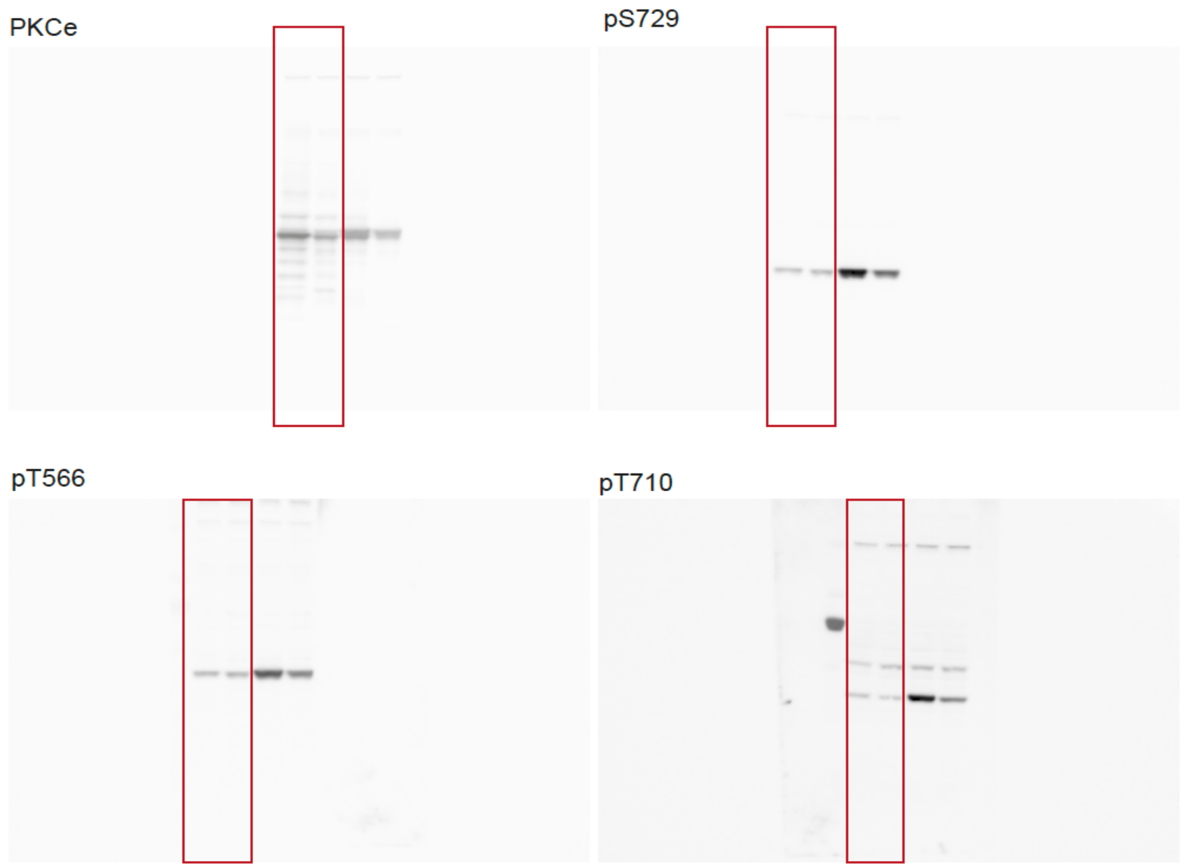

Supplementary Figure 2f

SERBP1

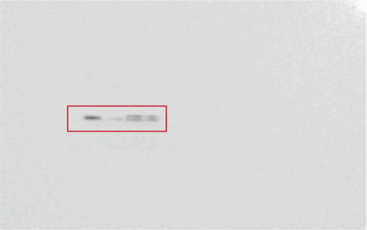

GAPDH

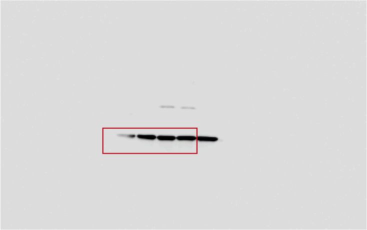

FLAG

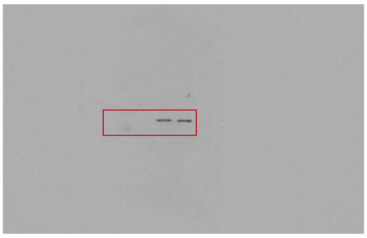

Supplementary Figure 2d

SERBP1

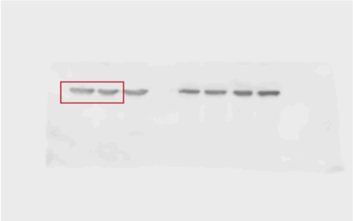

GAPDH

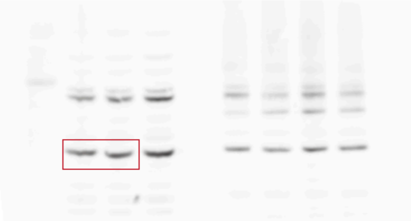

Supplementary Figure 2e

PKCε

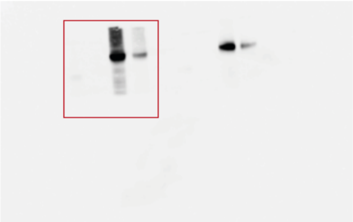

GAPDH

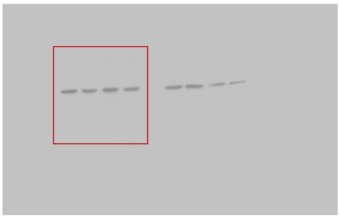

Supplementary Figure 2h

TopoIIα phospho-S29

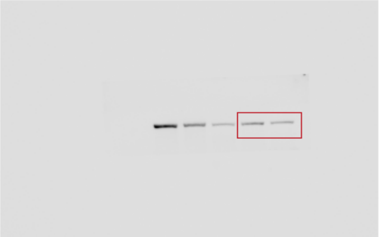

TopoIIα

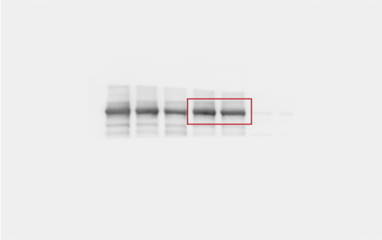

Supplementary Figure 2g

PKCε

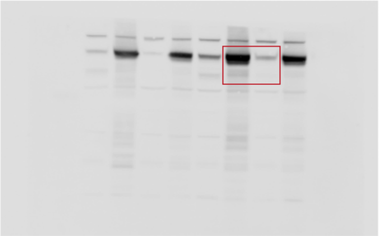

SERBP1

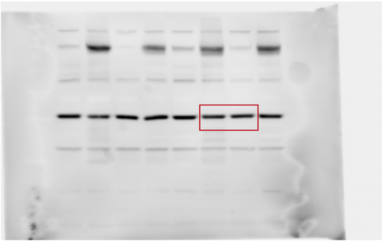

GAPDH

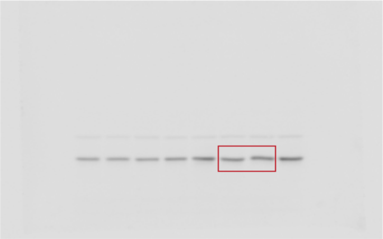

Supplementary Figure 3f

PKCε

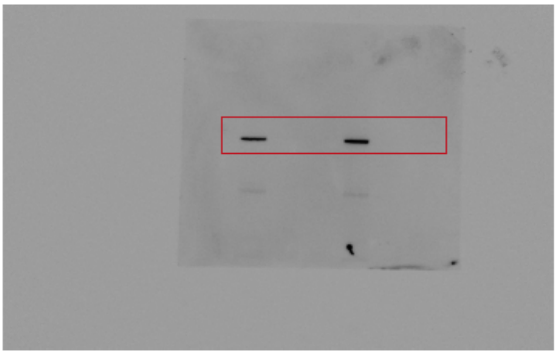

RPS6

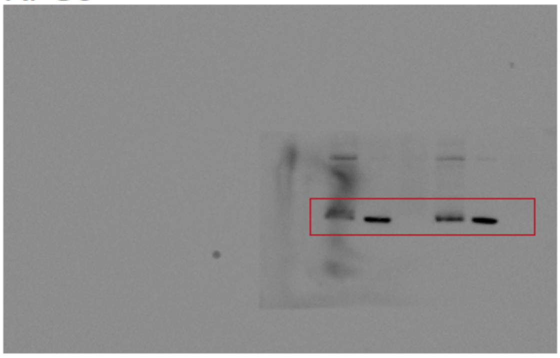

GM130

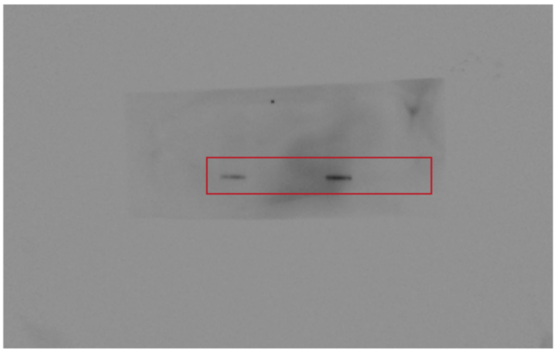

GAPDH

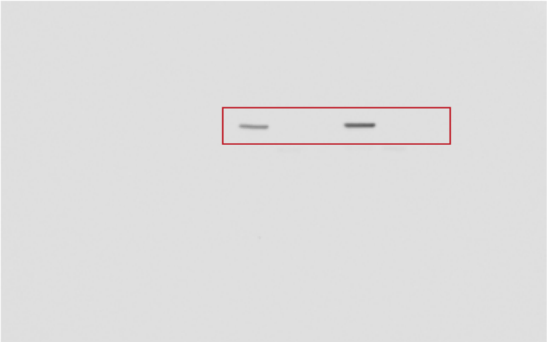

EGFR

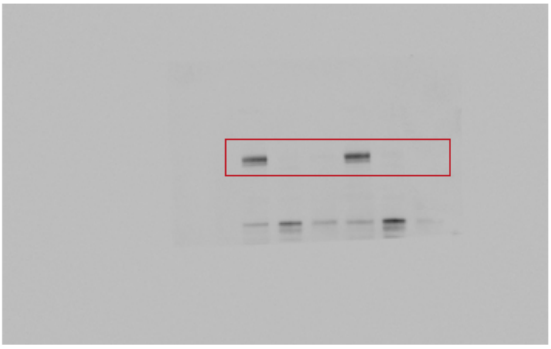

Vimentin

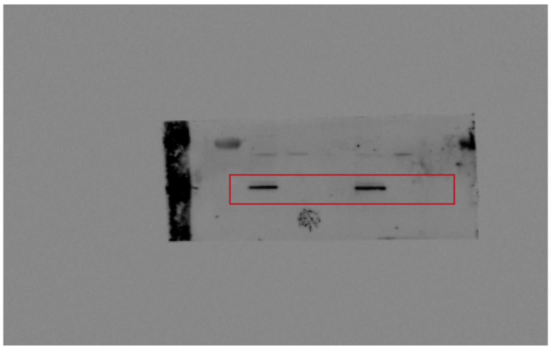

Supplementary Figure 3e

SERBP1

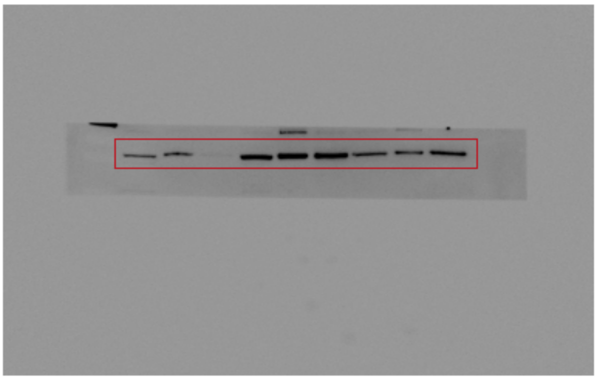

G3BP

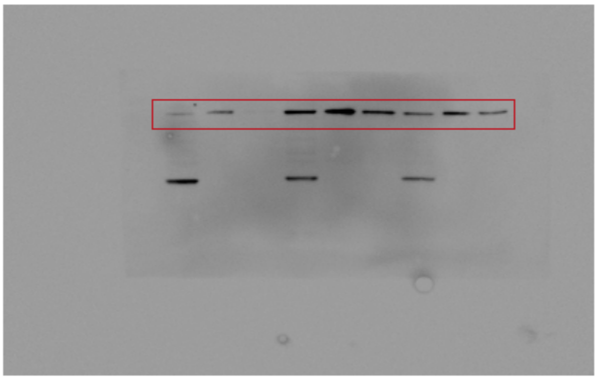

Supplementary Figure 5e

	siControl	siFMRP
EXP1	6.878307	11.47059
EXP2	6.390977	12.32227
EXP3	4.709141	8.282209
